# Contrasting global, regional and local patterns of genetic structure in gray reef shark populations from the Indo-Pacific region

**DOI:** 10.1038/s41598-019-52221-6

**Published:** 2019-11-01

**Authors:** E. Boissin, S. R. Thorrold, C. D. Braun, Y. Zhou, E. E. Clua, S. Planes

**Affiliations:** 1 PSL Research University: EPHE-UPVD-CNRS, USR 3278 CRIOBE, Université de Perpignan, 52 Avenue Paul Alduy, 66860 Perpignan, Cedex France; 2 Laboratoire d’Excellence CORAIL, Papetoai, French Polynesia; 3 Biology Department, Woods Hole Oceanographic Institution, Woods Hole, MA 02543 USA; 4 Massachusetts Institute of Technology—Woods Hole Oceanographic Institution Joint Program in Oceanography/Applied Ocean Science and Engineering, Cambridge, MA 02139 USA; 5 Present Address: School of Aquatic and Fishery Sciences, University of Washington, Seattle, WA 98195 USA

**Keywords:** Evolutionary ecology, Conservation biology

## Abstract

Human activities have resulted in the loss of over 90% of sharks in most ocean basins and one in four species of elasmobranch are now listed at risk of extinction by the IUCN. How this collapse will affect the ability of populations to recover in the face of continued exploitation and global climate change remains unknown. Indeed, important ecological and biological information are lacking for most shark species, particularly estimates of genetic diversity and population structure over a range of spatial scales. Using 15 microsatellite markers, we investigated genetic diversity and population structure in gray reef sharks over their Indo-Pacific range (407 specimens from 9 localities). Clear genetic differentiation was observed between the Indian and the Pacific Ocean specimens (F_ST_ = 0.145***). Further differentiation within the Pacific included a West and East cleavage as well as North-Central and South-Central Pacific clusters. No genetic differentiation was detected within archipelagos. These results highlight the legacy of past climate changes and the effects of large ocean expanses and circulation patterns on contrasting levels of connectivity at global, regional and local scales. Our results indicate a need for regional conservation units for gray reef sharks and pinpoint the isolation and vulnerability of their French Polynesian population.

## Introduction

Anthropogenic pressures, including over-fishing and habitat destruction, have resulted in the loss of over 90% of sharks and large predatory fishes across all ocean basins^1–3^. Collapse of shark populations has had profound effects on ecosystem functioning and resilience^4–6^ and potentially threatens their capacity to maintain demographic connectivity and genetic diversity across their ranges^7^. Reef sharks are of particular concern as they are a primarily reef-associated group that plays an important structural role in coral reef ecosystems as apex or meso-predators^8–10^. Recent work has reported striking declines in reef shark densities associated with the size of adjacent human populations^8^. More information on basic evolutionary and demographic processes are urgently needed to ensure conservation of individual reef shark populations and the ecosystem services that they provide across the world’s tropical and sub-tropical oceans^9,10^.

The application of molecular DNA techniques in conservation biology is well established^7^. Molecular tools are, in many cases, the only means available to estimate essential parameters for populations, and this is certainly the case for rare and elusive species. Genetic methods have been used to infer dispersal patterns, demographic trends and important reproductive traits in many sharks. Indeed, mitochondrial sequences and microsatellite markers have revealed genetic structure and recent declines in whale sharks^11^, a strong trans-Pacific break and the need for regional conservation units in the Galapagos shark^12^, and panmixia throughout the Indo-Pacific range of the highly migratory tiger shark^13^. Furthermore, reef shark studies have demonstrated significant genetic structure and variable demographic histories for blacktip reef sharks over their Indo-Pacific range^14^, and female blacktip reef sharks were shown to be philopatric in French Polynesia^15^. Genetic differentiation is also apparent in whitetip reef sharks across the Indo-Pacific but populations are more homogeneous at the regional scale^16^. Taken together, these studies illustrate that spatial and temporal variability in genetic differentiation among populations often reflect important components of the movement ecology of elasmobranch species.

The gray reef shark (*Carcharhinus amblyrhynchos*) is a reef-associated species distributed throughout the Indian Ocean and West and Central Pacific^17,18^. Gray reef sharks are among the most abundant reef sharks in the Indo-Pacific and can comprise up to 50% of the upper trophic level biomass on coral reefs in some areas^19^. Yet possible collapses of gray reef shark populations have been documented, even on reefs with relatively low human impacts^20^. Without a pelagic larval phase in their life cycle, population connectivity in gray reef sharks occurs exclusively through movements of adults and juveniles^21–24^. These movements and residency patterns are complex and vary among reefs and life stages and between sexes^22–24^. Gray reef sharks have been shown to change territory often at some locations (Rangiroa, French Polynesia^25^) while at other places they have been documented to stay up to two years within the same territory (the Great Barrier Reef^24,26^, GBR). Low residency was also recorded on the semi-isolated reefs of the Great Barrier Reef^23^ whereas on isolated habitats, gray reef sharks seem to exhibit longer residency on a single reef^27,28^. The few genetic studies undertaken to date on gray reef sharks have shown high migration frequency along the Great Barrier Reef^29^, a barrier to gene flow across large expanses of ocean, and an isolation-by-distance pattern (when the genetic distance between populations is proportional to the geographic distance between these populations) along the Australian continental shelf^30^. So far, no study has investigated the genetic composition of gray reef shark populations across the species’ range. Therefore, while reef characteristics such as geography and oceanographic context likely play an important role, more specific factors regulating dispersal of gray reef sharks remain unknown.

**Figure 1:**
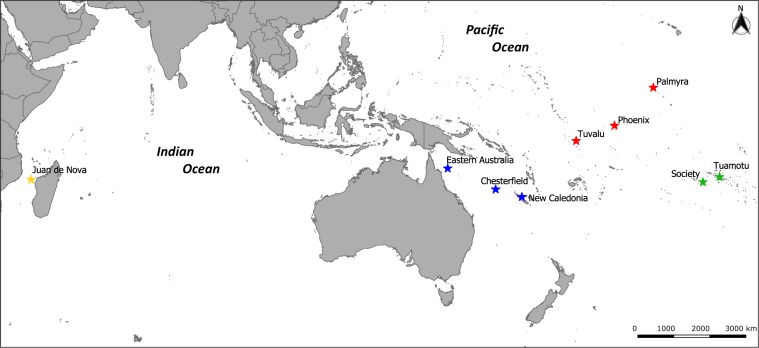
Map of the nine Indo-Pacific localities sampled for the 407 gray reef shark specimens analyzed in this study. The colors represent the genetic groups revealed in the analyses.

Here, we used 15 microsatellite markers to investigate the genetic structure and diversity of the gray reef shark populations from French Polynesia and the Line islands to the western Indian Ocean (Fig. 1; Table S1). We focused efforts on contrasting local and regional levels of gene flow in the Central and West Pacific. Population structure will likely be strongly influenced by behavioral trade-offs between an inherent ability to roam over large distances (high gene flow potential) while also displaying site-fidelity and residency behavior (low gene flow potential).

## Results

### Genetic diversity

Mean allele numbers ranged from 4.7 to 15.5 (Table 1). Private allele numbers per sampling site ranged from 0 to 18 while there was a total of 143 alleles found exclusively in the Pacific Ocean and 10 alleles recovered only from the western Indian Ocean. Observed and expected heterozygosity values ranged from 0.7120 to 0.8500 (Table 1). Inbreeding coefficients were significant for the Australian Great Barrier Reef, French Polynesia and the Phoenix archipelago samples (Table 1).

**Table 1:** Summary statistics of the nine sampling localities of gray reef sharks collected in the Indian and the Pacific Oceans.

		N	A	A_P_	H_o_	H_e_	F_is_
West Pacific	Eastern Australia (GBR)	21	10.2	9	0.7322	0.7927	0,078***
	Chesterfield	34	11.7	7	0.7976	0.7894	−0,010 ns
	New Caledonia	13	8.7	0	0.8205	0.8033	−0,022 ns
	Total	68	13.9	21	0.7820	0.7990	0.021 ns
North Central Pacific	Tuvalu	4	4.7	1	0.8500	0.7833	−0,100 ns
	Palmyra	50	12.1	4	0.7532	0.7681	0,020 ns
	Phoenix	164	15.5	18	0.7378	0.7778	0,052***
	Total	218	15.9	33	0.7434	0.7778	0.044***
South Central Pacific	Society	14	7.9	0	0.7143	0.7623	0,065**
	Tuamotu	74	11.5	2	0.7120	0.7582	0,061***
	Total	88	11.7	3	0.7124	0.7598	0.063***
	Total Pacific Ocean	374	17.73	143	0.7431	0.7848	0.053***
Indian Ocean	Mozambique Channel	33	8.9	10	0.7152	0.7302	0,021 ns

N = number of specimens analyzed; A = Mean number of alleles; A_P_ = Number of private alleles; H_o_ = observed heterozygosity; He = non-biased expected heterozygosity; F_is_ = inbreeding coefficient and its significance: ns = non-significant, **significant at p < 0.01 and ***at p < 0.001.

### Genetic differentiation between ocean basins

**Figure 2:**
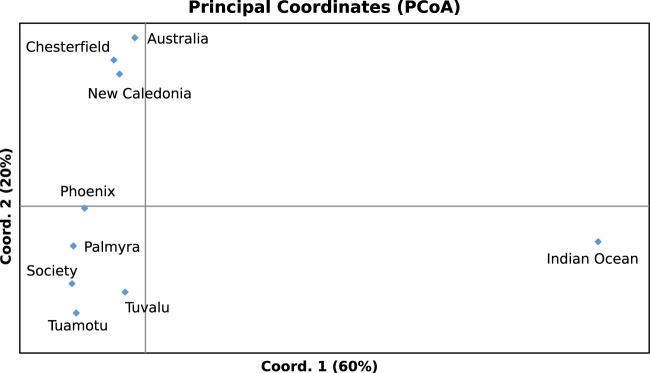
Principal Coordinates Analysis on the gray reef shark samples genotyped at 15 microsatellite loci.

**Figure 3:**
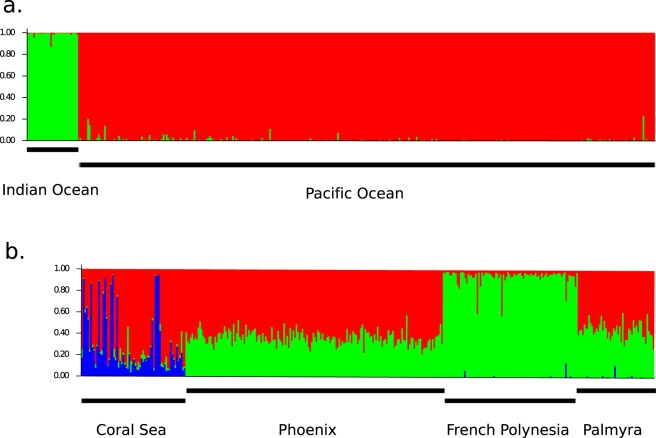
Bayesian Structure plots showing the most likely number of clusters (K) partitioning the dataset when including (**a**) all 407 specimens from the Indian and the Pacific Ocean, K = 2; and (**b**) the Pacific specimens only, K = 3. The most likely number of clusters were determined using the Evanno’s method (Evanno *et al*. 2005).

The F_st_ pairwise comparison between western Indian Ocean and Pacific Ocean populations was high and significant (F_st_ = 0.145***). Therefore, all individual F_st_ values including the Indian Ocean were significant and high (from 0.145 to 0.161, Table 2). Furthermore, the first axis of the Principal Coordinates Analysis (PCoA) clearly differentiated the western Indian Ocean samples from the samples collected in the Pacific Ocean (Fig. 2). This differentiation was also clear in the Bayesian clustering analysis which suggested two major clusters between the western Indian and Pacific basins (K = 2; Fig. 3a). Finally, the test of isolation-by-distance, when considering the entire dataset, was significant (p < 0.001).

**Table 2:** Pairwise F_st_ values among the nine samples of the gray reef sharks from the Indo-Pacific.

	Chesterfield	New Caledonia	Mozambique Channel	Palmyra	Phoenix archipelago	Society	Tuamotu	Tuvalu
Eastern Australia	0.018***	0.020**	0.150***	0.042***	0.033***	0.040***	0.047***	0.036*
Chesterfield		−0.004 ns	0.148***	0.025***	0.015***	0.025***	0.034***	0.037*
New Caledonia			0.145***	0.019***	0.012**	0.026**	0.032***	0.035*
Mozambique Channel				0.160***	0.150***	0.161***	0.160***	0.146**
Palmyra					0.005***	0.009*	0.013***	0.022 ns
Phoenix						0.009**	0.012***	0.018 ns
Society							0.003 ns	0.019 ns
Tuamotu								0.019*

Significance is given as follows: ns = non-significant, *p-value < 0.05, **p-value < 0.01, ***p-value < 0.001.

### Genetic differentiation within the Pacific Ocean

Of the total 45 pairwise F_st_ comparisons only 5 were not significant, and those were always between close localities such as New Caledonia *vs*. Chesterfield, Society *vs*. Tuamotu, or for localities with few specimens (Tuvalu N = 4, Table 2). Regarding the PCoA, the second axis clearly separated West Pacific samples (Australia, Chesterfield and New Caledonia) and Central Pacific samples (Tuvalu, Phoenix, Palmyra and French Polynesia, Fig. 2). The Bayesian clustering, focusing on Pacific specimens only, also revealed Central and West Pacific groups but showed further segregation within the Central Pacific samples: Phoenix, Tuvalu and Palmyra grouped together while French Polynesia was separated in another group with a distinct genetic composition (Fig. 3b). At the scale of an archipelago (Phoenix) or two close archipelagos (Society and Tuamotu), few pairwise F_st_ comparisons were significant (Supp Data Tables S2, S3). Finally, the test of isolation-by-distance was also significant when considering the Pacific Ocean samples only (i.e. removing the western Indian Ocean samples), with or without Tuvalu (N = 4; p < 0.01 and p < 0.001, respectively).

## Discussion

Population genetic structure of gray reef sharks throughout their Indo-Pacific range revealed contrasting levels of connectivity across global, regional and local scales. The effects of the Indo-Pacific barrier are clear between the Indian and the Pacific Ocean samples. Gray reef sharks also showed regional genetic differentiation between the West and Central Pacific, likely generated by the large expanses of open water between the regions. We also found some level of genetic differentiation between the North and South-Central Pacific, suggesting potential effects of oceanic current circulation on the population structure of gray reef sharks. We found no significant genetic differentiation on small spatial scales within the Phoenix archipelago or within French Polynesia. However, we detected a pattern of isolation-by-distance among populations of both oceans and within the Pacific. Overall, the genetic structure found in gray reef sharks appears higher than that of reef fishes, suggesting that adult movements of this shark limit dispersal capabilities compared to the pelagic larval stage of teleost coral reef fishes^31^.

The western Indian Ocean population of the gray reef shark is clearly separated from the rest of the samples collected in the Pacific Ocean (Table 2, Figs 2 and 3A). Momigliano *et al*.^30^ also demonstrated genetic differentiation between the Pacific Ocean samples of gray reef sharks and samples from the Chagos Archipelago in the North Indian Ocean. However, more samples in the northern and the eastern Indian Ocean are needed to pinpoint the precise area of the genetic break between Indian and Pacific populations. Significant genetic differentiation between Indian and Pacific oceans populations has also been identified in many fish species^32,33^, including three other coastal shark species. The scalloped hammerhead shark *Sphyrna lewini*^34^, the blacktip shark *Carcharhinus limbatus*^35^ and the whitetip shark *Triaenodon obesus*^36^ all demonstrated a significant genetic break across the Indo-Pacific barrier; however, neither tiger sharks^13^ or whale sharks^11^ show a similar discontinuity across this barrier. The most likely explanation for the genetic break in reef fish, including sharks, is the effect of the low sea-level stands during glacial periods (low sea-level, about 100 m from current level, during glacial periods)^33,36^. Moreover, the large oceanic distances are now likely hindering widespread genetic exchange between the gray reef shark populations of each ocean basin as large stretches of deep open water have been suggested to create a barrier to gene flow for this species^30^. The strong genetic differentiation that we found (Table 2, Figs 2 and 3A) is even more noteworthy as sharks have slow rates of evolution compared to mammals and bony fish due to their slow metabolic rates^37^. From the current sampling, it is not clear if these populations are still capable of interbreeding or if they should be considered as distinct sister species given the numerous private alleles (Table 1). For the Indo-West Pacific marine biogeographic area, a contact zone is known to be located at Christmas and Cocos (Keeling) islands for many reef fishes^38^. The Cocos population from Momigliano *et al*.^30^ seems to show a mixed genetic make-up but future gray reef shark sampling should focus on this area and on northern areas in Indonesia to definitively assess the status of the Indian and the Pacific populations (i.e. sister species or intraspecific genetic differentiation).

At a regional scale, gray reef shark samples from the Pacific Ocean were clustered in distinct groups, with the Coral Sea specimens forming one cluster (Eastern Australia, Chesterfield and New Caledonia), the North-Central specimens forming another cluster (Tuvalu, Phoenix and Palmyra) and the South-Central specimens (French Polynesia) comprising a third cluster (Fig. 3b). Additionally, Tuvalu, Phoenix and Palmyra populations were more closely related to each other than Palmyra and French Polynesia (Fig. 3b), whereas the Line islands (Palmyra) and French Polynesia are geologically and geographically linked with the intersection of the Line islands seamount chain with the Tuamotu plateau. Genetic differentiation has previously been reported between Central and West Pacific teleost (bony) reef fish populations^39,40^ as well as in the sedentary whitetip sharks^16^. Large ocean expanses between these two regions likely reduce the probability of long-distance dispersal. Schultz *et al*.^41^ revealed moderate genetic differentiation (F_ST_ = 0.07) between Australian and French Polynesian populations of the Sicklefin lemon shark *Negaprion acutidens* and proposed a stepping stone mechanism for rare long-distance dispersal events between West and Central Pacific islands. Additionally, genetic differentiation between the Line Islands and French Polynesia has been demonstrated in the three-spot damselfish, *Dascyllus trimaculatus*^42^ and the brown surgeonfish *Acanthurus nigrofuscus*^43^. This genetic differentiation on either side of the equator in the Central Pacific likely arose due to isolation maintained by the major currents in the zone (Equatorial Counter Current and North Equatorial Current)^44,45^. A similar pattern of genetic differentiation in gray reef sharks suggests that dispersal seems to be dependent, to some extent, on the ocean circulation and the major currents in the area likely limit migration of sharks across this boundary. Gray reef sharks are viviparous and therefore, contrary to most reef fishes, population connectivity occurs through movements of adults and juveniles rather than dispersal of eggs and larvae. It is therefore likely that the same barriers to dispersal are acting for species both with and without larval dispersal. However, gray reef sharks have strong and complex reef residency behaviors^23,24^ that, when combined with the large expanse of deep open ocean between the regions, apparently reduces the frequency of long-distance movements. Thus, the genetic structure of gray reef sharks appears somewhat higher than that of reef fishes, that are more homogenous across their range^39,42,43^. We did identify a pattern of isolation-by-distance in our dataset when including all samples, samples from the Pacific only, and when removing the small size sample (Tuvalu), indicating that dispersal probability in gray reef sharks is proportional to geographic distance among populations. Similar patterns of isolation-by-distance were described by Momigliano *et al*.^30^ and highlight the low probability of adult gray reef sharks migrating long distances across expanses of open ocean.

At a local scale, we collected sufficient numbers of samples to investigate genetic differentiation at the scale of a single archipelago (Phoenix archipelago, about 500 km in diameter) or several close archipelagos (French Polynesia, 500 km to 1000 km). No significant differences among reefs were found in either comparison. Momigliano *et al*.^29^ similarly detected no genetic differentiation in gray reef sharks over the 1200 km of the Great Barrier Reef system. In contrast, blacktip reef sharks show differentiation both among and within the archipelagos of French Polynesia^14,46^. Blacktip reef sharks must, therefore, either home to natal reefs at much higher frequencies, or move among reefs at much lower frequencies, than gray reef sharks. Interestingly, inbreeding coefficients from Eastern Australia, Phoenix, Society and Tuamotu samples were significant, suggesting somewhat limited exchange among reefs that went undetected by more traditional statistical analyses based on F_st_. Overall, our results suggest that a small number of gray reef sharks make infrequent long-distance movements between reefs across open water. White *et al*.^28^ came to a similar conclusion based on a small sample of satellite-tagged gray reef sharks at Palmyra Atoll in the North tropical Pacific. Two from a total of six sharks were detected outside of the 200 NM limit MPA (Marine Protected Area) boundary surrounding Palmyra Atoll for 3% and 46% of their daily location estimates. The shark that traveled the furthest reached a maximum linear distance of 908 km from the 12NM MPA boundary surrounding Palmyra Atoll^28^.

The need to consider genetic diversity in conservation of sharks was recently re-emphasized^7,47^. In our study, observed heterozygosity values were comparable for all the samples analyzed and also to those from the gray reef sharks of the Great Barrier Reef (0.79 to 0.8^29^). These values were higher than in blacktip reef sharks^28^, where observed heterozygosity ranged from 0.47 to 0.67 but comparable to that of other shark species also analyzed with microsatellite markers^7^. Noticeably, heterozygosity levels did not correlate with the level of isolation of a reef, as all reefs show similar values. Thus, we found no evidence for recent genetic bottlenecks, as suggested for other reef shark species^28^. In our study, we further revealed clear genetic groupings across the Pacific region, with genetic differentiation among broader geographic clusters (West vs Central Pacific and North vs South Central Pacific; Fig. 3b). Gray reef sharks should thus be divided into regional conservation units, as are many marine mammals and sea turtles^48,49^. Similarly, 3 to 4 conservation units were recently suggested for the Galapagos sharks across their Pacific range^12^. For reef sharks, a strong tendency for site fidelity and residency behavior should also be taken into consideration when delineating conservation units. However, at the scale of an archipelago (Phoenix) or several closely related archipelagos (French Polynesia), gray reef sharks appear to represent quasi-panmictic units with free movements of individuals across reefs. Juhel *et al*.^50^ also showed that human-linked behavioral alterations should be considered in management strategies to ensure the persistence of gray reef shark populations. Furthermore, rare long-distance dispersal across open ocean barriers seem to be an important process driving genetic diversity and structure in gray reef sharks. Open ocean movements through “high-seas” corridors therefore represent an important component of the life history of at least some coastal sharks that functions to reduce local inbreeding. “High-seas” are currently the subject of much debate at the United Nations, and the first-ever “high-seas” conservation treaty is expected to be finalized in 2020. Momigliano *et al*.^28^ went so far as to suggest a move from discontinuous networks of MPAs on the GBR to MPAs connected via protected corridors to better protect these sharks with larger home ranges. A conservation plan for these gray reef shark populations should therefore include several conservation units with the potential for migration corridors in the high seas that would serve to connect regional reefs within each conservation unit. The Indian Ocean gray reef sharks clearly represent a conservation unit on their own while another conservation unit can be identified for the West Pacific and two others for the North and South-Central Pacific populations (Fig. 3a,b).

Finally, French Polynesia appeared isolated from the rest of the gray reef shark populations including archipelagos in the Central Pacific (Fig. 3b). The isolation of the French Polynesian atolls has been demonstrated in several species, with a complex legacy of past climate effects and rare contemporaneous migrations^43,51^. This genetic isolation has direct implications for conservation and resilience of sharks in the region and French Polynesia should be considered as a separate conservation unit. Blacktip reef sharks also showed signals of recent bottleneck in Moorea, likely due to the growing anthropogenic pressure in these populated islands^14^. Furthermore, gray reef sharks in the Tuamotu archipelago seem to show peculiarities, suggesting that this population may warrant a high conservation priority. Indeed, their extremely high number at Fakarava Atoll was recently demonstrated to produce an inverted trophic pyramid, maintained through subsidies linked to spawning aggregations of teleosts^52^. Finally, as French Polynesia was recently shown as a likely past glacial refuge for green sea turtles^53,54^ and as a projected refuge under future warm conditions^55,56^, the gray reef sharks from this region are particularly important in a conservation context as they represent a genetically-distinct group and are likely to be less impacted by warmer conditions in this refuge.

## Material and Methods

### Ethical statement

All samples were collected in agreement with local legislations. No permit was required for DNA collection in New Caledonia. Research on sharks in French Polynesia was approved under Arrêté N° 9524 issued by the Ministère de la Promotion des Langues, de la Culture, de la Communication et de l’Environnement of the French Polynesian government on 30 October 2015, and Arrêté N° 5129 issued by the Ministère de la Promotion des Langues, de la Culture, de la Communication et de l’Environnement of the French Polynesian government on 22 June 2016. Samples in the Phoenix Islands Protected Area were collected under relevant permits from the Government of Kiribati and sampling protocols approved by Woods Hole Oceanographic Institution Institutional Animal Care and Use Committee (IACUC) ID number 18417. Sampling protocols for Palmyra Atoll collections were certified by the IACUC, University of California, Santa Barbara, Protocol no. 856 (date of IACUC approval: 5/31/2012) under U.S. Fish and Wildlife Service special use permits (Permit Numbers #12533–14011, #12533–13011, #12533–12011, #12533–11007, #12533–10011, #12533–09010, #12533–08011, and #12533–07006). Samples from the Great Barrier Reef were provided by collaborators from Australia in accordance with local regulations.

### Sample collection

A total of 407 sharks were sampled at 9 locations in the South West Indian Ocean, and the West and Central Pacific (Fig. 1, see Table S1 for details). Fin clips were collected from sharks sampled by hooks and lines. Sharks were brought to the surface and put in tonic immobility along the side of small boats and restrained with a tail line while cutting a small tissue sample from one of the fins and then immediately released.

### Molecular data

Total DNA was extracted from fin clips using the PureGene protocol (Qiagen, Hilden, Germany). A total of 15 microsatellite markers developed for gray reef sharks were amplified using the primers and cycling parameters from Momigliano *et al*.^57^. PCR products were sent for sequencing to an external private company (GenoScreen, Lille, France) to be run on an Applied Biosystems 3730 sequencer and were genotyped employing GeneMapper software version 4.0 (Applied Biosystems, Foster City, CA, USA).

### Data analyses

The presence of null alleles, scoring errors and large allele drop-out was verified using Micro-checker v2.2.3^58^. The mean number of alleles (A), observed (Ho) and expected (He) heterozygosity, the inbreeding coefficient (F_is_) and the fixation index (F_st_) were computed in Genetix v4.05.02^59^. The number of private alleles (A_p_) was computed in GenAlEx v6.5^60^. The correction for multiple tests of Benjamini & Hochberg^61^ was applied.

To investigate population structure, a Principal Coordinates Analysis (PCoA) on populations was first computed in GenAlEx. Additionally, Structure v2.3^62^ was used to search for the most likely number of clusters. This analysis was run with no priors by location. After initial runs, the parameters were set as follows: a burn-in period of 100 000 iterations followed by 500 000 recorded iterations for K = 1 to K = 9 clusters and 15 iterations per K values. We ran a first analysis with all specimens and a second analysis with the specimens from the Pacific Ocean only. In this last analysis, we used sampling locations as prior (model LOCPRIOR^63^), known to be able to reveal subtle differentiations. The most probable number of clusters present in this dataset was determined using the Evanno’s *Δ*K approach^64^ and computed with Structure Harvester online^65^.

We also tested for  a pattern of isolation-by-distance using a Mantel test in Genetix between the matrix of genetic distances [F_st_ ⁄ (1 – F_st_)]^66^ and the matrix of log coastal geographical distances (in km) of each pair of localities. Significance was tested using a random permutation procedure implemented in Genetix (5 000 permutations). This analysis was performed on the whole dataset and on the Pacific samples only with or without the site with small sample size (Tuvalu, N = 4).

## Supplementary information

Supplementary Tables

Table S4

## Data Availability

The dataset consisting of 407 multilocus genotypes is provided in Table S4.
