# Supplementary material for: Contrasting global, regional and local patterns of genetic structure in gray reef shark populations from the Indo-Pacific region
Source: Sci Rep. 2019 Nov 1;9:15816. doi: 10.1038/s41598-019-52221-6 (PMC6825237; doi:10.1038/s41598-019-52221-6)
Supplement: Supplementary file 1 — Supplementary Tables [file 41598_2019_52221_MOESM1_ESM.docx]

Contrasting global, regional and local patterns of genetic structure in gray reef shark populations from the Indo-Pacific region

Boissin E, Thorrold SR, Braun CD, Zhou Y, Clua E, Planes S

Table S1. Details of sampling locations

| **Ocean** | **Country/Archipelago** | **Date** | **Locality** | **N** |
| --- | --- | --- | --- | --- |
| Indian | Mozambique Channel | 2009 | Zélée Bank | 7 |
|  |  |  | Juan de Nova | 26 |
| Pacific | Chesterfield | Nov. 2011 | Chesterfield | 12 |
|  |  |  | Bampton | 13 |
|  |  |  | Passage islet | 2 |
|  |  |  | Avon isles | 7 |
|  | New Caledonia | March 2010 | Belep | 8 |
|  |  | July 2010 | Poindimié | 5 |
|  | Eastern Australia | 2010 | Osprey Reef | 21 |
|  | Society | 2015 | Moorea | 9 |
|  |  | April 2017 | Tahiti | 5 |
|  | Tuamotu | 2007 | Nengo | 6 |
|  |  | 2007 | Faaite | 5 |
|  |  | June 2011 | Fakarava | 19 |
|  |  | 2014 | Tapoto | 2 |
|  |  | March 2017 | Tikehau | 7 |
|  |  | March 2017 | Apataki | 25 |
|  |  | Feb 2016 | Faaite | 5 |
|  |  | March 2009 | Acteon | 5 |
|  | Line Islands | 2017 | Palmyra | 50 |
|  | Phoenix | July2014/Sept 2015 | Birnie | 17 |
|  |  |  | Enderbury | 21 |
|  |  |  | Kanton | 27 |
|  |  |  | Manra | 2 |
|  |  |  | McKean | 24 |
|  |  |  | Niku | 44 |
|  |  |  | Orona | 12 |
|  |  |  | Rawaki | 11 |
|  |  |  | Winslow | 6 |
|  | Tuvalu | 2014 |  | 4 |
|  |  |  |  |  |
|  |  |  |  |  |

Table S2. Pairwise F_st_ comparisons between islands of the Phoenix archipelago. The number of specimens analyzed are indicated between brackets. Significance of P-values: *>0.05; **>0.01

| F_st_ | Enderbury (21) | Kanton (27) | Manra (2) | McKean (24) | Niku (44) | Orona (12) | Rawaki (11) | Winslow (6) |
| --- | --- | --- | --- | --- | --- | --- | --- | --- |
| Birnie (17) | 0.00117 | -0.00330 | -0.01625 | -0.00038 | 0.00337 | 0.00389 | 0.01259* | -0.00573 |
| Enderbury (21) |  | 0.00098 | 0.01457 | 0.00361 | 0.00206 | -0.00196 | 0.01694** | -0.00491 |
| Kanton (27) |  |  | 0.00252 | 0.00242 | -0.00307 | -0.00766 | 0.00878 | -0.00616 |
| Manra (2) |  |  |  | 0.02727 | 0.01045 | 0.02078 | 0.01516 | -0.02299 |
| McKean (24) |  |  |  |  | 0.00332 | 0.00578 | 0.01636** | 0.00515 |
| Niku (44) |  |  |  |  |  | -0.00328 | 0.00982* | -0.00736 |
| Orona (12) |  |  |  |  |  |  | 0.00827 | -0.00330 |
| Rawaki (11) |  |  |  |  |  |  |  | 0.00472 |

Table S3. Pairwise F_st_ comparisons for French Polynesia islands. The number of specimens analyzed are indicated between brackets. Significance of P-values: *>0.05

| F_st_ | Faaite (10) | Acteon (4) | Nengo (4) | Moorea (9) | Tahiti (5) | Tikehau (7) | Fakarava (20) | Nord (4) |
| --- | --- | --- | --- | --- | --- | --- | --- | --- |
| Apataki (25) | 0.01354* | -0.00057 | 0.00798 | 0.00793 | -0.00473 | 0.00231 | 0.00738 | 0.00492 |
| Faaite (10) |  | 0.02242 | -0.00031 | 0.01792 | -0.00233 | 0.03257 | 0.01776 * | 0.00612 |
| Acteon (4) |  |  | -0.00343 | -0.01862 | -0.00127 | 0.01907 | 0.00689 | 0.00884 |
| Nengo (4) |  |  |  | 0.00612 | -0.00089 | 0.03600 | 0.00704 | -0.01912 |
| Moorea (9) |  |  |  |  | -0.00053 | 0.02415 | 0.00568 | 0.00642 |
| Tahiti (5) |  |  |  |  |  | 0.00845 | 0.02111 | -0.00920 |
| Tikehau (7) |  |  |  |  |  |  | 0.01518 | 0.02004 |
| Fakarava (20) |  |  |  |  |  |  |  | -0.00300 |
