# Supplementary material for: Contrasting global, regional and local patterns of genetic structure in gray reef shark populations from the Indo-Pacific region
Source: Sci Rep. 2019 Nov 1;9:15816. doi: 10.1038/s41598-019-52221-6 (PMC6825237; doi:10.1038/s41598-019-52221-6)
Supplement: Supplementary file 2 — Table S4 [file 41598_2019_52221_MOESM2_ESM.docx]

Contrasting global, regional and local patterns of genetic structure in gray reef shark populations from the Indo-Pacific region

Boissin E, Thorrold SR, Braun CD, Zhou Y, Clua E, Planes S

Table S4. Multilocus genotypes of the 407 gray reef shark specimens analyzed at 15 microsatellite loci.

|  |  |  | mix 1 | | | | | | mix 2 | | | | | | | mix 3 | | | | |
| --- | --- | --- | --- | --- | --- | --- | --- | --- | --- | --- | --- | --- | --- | --- | --- | --- | --- | --- | --- | --- |
| Region | Localty | Sample Name | Camb 02 | Camb 07 | Camb 09 | Camb 11 | Camb 06 | Camb 22 | | Camb 20 | Camb 03 | Camb 18 | Camb 25 | Camb 04 | Camb 05 | | Camb 15 | Camb 27 | Camb 28 |  |
| Coral Sea | Australia | EstAus01 | 330338 | 304308 | 216218 | 173185 | 252261 | 352352 | | 327327 | 215237 | 159161 | 199199 | 345353 | 270278 | | 212216 | 173173 | 301303 |  |
| Coral Sea | Australia | EstAus02 | 322322 | 304308 | 212218 | 173187 | 246252 | 352352 | | 311327 | 207237 | 141151 | 195211 | 345353 | 278280 | | 210214 | 171175 | 285297 |  |
| Coral Sea | Australia | EstAus03 | 342342 | 304308 | 216218 | 173195 | 252261 | 352352 | | 323327 | 213237 | 139147 | 199223 | 345353 | 270278 | | 206208 | 173173 | 301301 |  |
| Coral Sea | Australia | EstAus04 | 330330 | 304308 | 218218 | 173187 | 252267 | 352352 | | 319321 | 207217 | 139151 | 199211 | 345353 | 278280 | | 208210 | 175179 | 297301 |  |
| Coral Sea | Australia | EstAus05 | 318318 | 288296 | 212214 | 173173 | 252252 | 352352 | | 313335 | 245245 | 151153 | 223223 | 345345 | 270280 | | 206210 | 163173 | 307307 |  |
| Coral Sea | Australia | EstAus06 | 322362 | 296312 | 212216 | 171175 | 252252 | 352352 | | 319323 | 229265 | 165179 | 199223 | 345361 | 276278 | | 194208 | 171173 | 297297 |  |
| Coral Sea | Australia | EstAus07 | 330330 | 312312 | 212212 | 171171 | 252252 | 352352 | | 327327 | 295295 | 139139 | 195195 | 345345 | 276276 | | 206214 | 171171 | 301301 |  |
| Coral Sea | Australia | EstAus09 | 346362 | 300308 | 214216 | 173173 | 246246 | 352352 | | 317327 | 233243 | 149173 | 191191 | 351353 | 266276 | | 206210 | 167173 | 297301 |  |
| Coral Sea | Australia | EstAus10 | 334342 | 308308 | 218218 | 173187 | 240261 | 352352 | | 315323 | 207223 | 147157 | 211223 | 349357 | 270278 | | 210222 | 171175 | 301301 |  |
| Coral Sea | Australia | EstAus12 | 330350 | 304312 | 218218 | 165187 | 240258 | 352352 | | 321333 | 207231 | 159185 | 199199 | 345359 | 270276 | | 200204 | 167171 | 297303 |  |
| Coral Sea | Australia | EstAus13 | 326338 | 304316 | 216220 | 173173 | 252255 | 352352 | | 311331 | 207257 | 139179 | 207215 | 353355 | 266278 | | 204206 | 155175 | 297301 |  |
| Coral Sea | Australia | EstAus14 | 000000 | 304304 | 212216 | 169173 | 252261 | 352352 | | 313327 | 233233 | 139179 | 223223 | 345361 | 268268 | | 202206 | 151175 | 285285 |  |
| Coral Sea | Australia | EstAus15 | 334362 | 304308 | 218218 | 173187 | 252255 | 352356 | | 313321 | 239259 | 143173 | 191199 | 345367 | 272282 | | 202212 | 171177 | 285301 |  |
| Coral Sea | Australia | EstAus17 | 346358 | 296308 | 212216 | 165187 | 252261 | 352352 | | 311331 | 223229 | 155163 | 211223 | 345371 | 276278 | | 212216 | 171171 | 301307 |  |
| Coral Sea | Australia | EstAus19 | 342342 | 304308 | 214214 | 169173 | 246252 | 352352 | | 313325 | 213237 | 139161 | 211211 | 345367 | 272278 | | 204212 | 141175 | 301303 |  |
| Coral Sea | Australia | EstAus20 | 326342 | 308308 | 212222 | 173175 | 246252 | 352352 | | 309313 | 213237 | 139161 | 191195 | 345353 | 266266 | | 210210 | 175187 | 285285 |  |
| Coral Sea | Australia | EstAus21 | 322334 | 304308 | 212216 | 171171 | 258270 | 352352 | | 321327 | 217241 | 139165 | 191195 | 349353 | 270272 | | 194212 | 167167 | 285297 |  |
| Coral Sea | Australia | EstAus26 | 342374 | 300304 | 216218 | 171195 | 240261 | 352352 | | 319327 | 237247 | 163167 | 199211 | 355355 | 266278 | | 198200 | 167167 | 299305 |  |
| Coral Sea | Australia | EstAus27 | 342342 | 300304 | 216216 | 173197 | 249270 | 352352 | | 323325 | 207217 | 147159 | 223227 | 353353 | 270272 | | 208216 | 173181 | 295301 |  |
| Coral Sea | Australia | EstAus28 | 326354 | 304308 | 212218 | 173187 | 246270 | 342352 | | 309321 | 207237 | 139157 | 195211 | 349361 | 266278 | | 202206 | 167173 | 285299 |  |
| Coral Sea | Australia | EstAus30 | 330354 | 300308 | 212214 | 173187 | 252261 | 342352 | | 309321 | 245245 | 139139 | 195195 | 345367 | 266266 | | 202208 | 173173 | 285301 |  |
| Coral Sea | Chesterfield | Bampton01 | 354354 | 304308 | 216218 | 183197 | 252261 | 352352 | | 311333 | 231241 | 175183 | 211223 | 345369 | 270270 | | 208210 | 173173 | 287297 |  |
| Coral Sea | Chesterfield | Bampton02 | 330346 | 304304 | 216218 | 171209 | 261261 | 352352 | | 311319 | 207207 | 147169 | 191191 | 353361 | 270278 | | 206206 | 165175 | 299301 |  |
| Coral Sea | Chesterfield | Bampton03 | 326350 | 304304 | 214216 | 173173 | 255261 | 352352 | | 323325 | 207217 | 151179 | 211223 | 349353 | 274278 | | 210210 | 167175 | 297305 |  |
| Coral Sea | Chesterfield | Bampton04 | 338378 | 300304 | 216218 | 173173 | 252252 | 352352 | | 311341 | 223227 | 159181 | 223227 | 345353 | 278280 | | 206210 | 173175 | 295301 |  |
| Coral Sea | Chesterfield | Bampton05 | 342346 | 300308 | 216218 | 173189 | 246255 | 352352 | | 311315 | 207213 | 183183 | 227227 | 345353 | 278280 | | 204206 | 167169 | 301301 |  |
| Coral Sea | Chesterfield | Bampton07 | 334354 | 308308 | 218218 | 171173 | 252258 | 352352 | | 315329 | 207241 | 163177 | 223227 | 345371 | 272278 | | 210214 | 167173 | 295301 |  |
| Coral Sea | Chesterfield | Bampton08 | 322350 | 304304 | 218218 | 173195 | 252258 | 352352 | | 311321 | 215245 | 163171 | 223227 | 345353 | 278280 | | 210210 | 165175 | 295297 |  |
| Coral Sea | Chesterfield | Bampton09 | 354370 | 304304 | 218220 | 171173 | 243249 | 352354 | | 303311 | 247265 | 151167 | 223235 | 345345 | 270278 | | 204206 | 167171 | 297301 |  |
| Coral Sea | Chesterfield | Bampton10 | 338362 | 304308 | 216218 | 175189 | 246255 | 352356 | | 325335 | 219251 | 151173 | 211227 | 349353 | 270276 | | 204218 | 165173 | 297297 |  |
| Coral Sea | Chesterfield | Bampton11 | 338350 | 300308 | 216218 | 165189 | 249261 | 352352 | | 327331 | 209239 | 151177 | 211223 | 353363 | 278280 | | 204206 | 173175 | 295297 |  |
| Coral Sea | Chesterfield | Bampton12 | 342342 | 304312 | 216218 | 173179 | 252252 | 352352 | | 321325 | 241269 | 145183 | 223227 | 345353 | 272280 | | 206210 | 167173 | 297301 |  |
| Coral Sea | Chesterfield | Bampton13 | 342370 | 304304 | 216218 | 181183 | 246258 | 352354 | | 331331 | 207247 | 165181 | 199227 | 353359 | 270278 | | 200206 | 169175 | 297303 |  |
| Coral Sea | Chesterfield | AvondN01 | 334354 | 304304 | 216216 | 165173 | 258261 | 352352 | | 321327 | 207217 | 177179 | 227235 | 345353 | 270270 | | 194200 | 175175 | 293301 |  |
| Coral Sea | Chesterfield | AvondN02 | 342342 | 304308 | 216218 | 173181 | 234270 | 352354 | | 319331 | 215231 | 151171 | 199227 | 345345 | 270282 | | 202206 | 167179 | 297301 |  |
| Coral Sea | Chesterfield | AvondN03 | 326346 | 300304 | 216218 | 183189 | 243252 | 352356 | | 329331 | 237253 | 157179 | 223223 | 359361 | 266272 | | 206208 | 151173 | 297303 |  |
| Coral Sea | Chesterfield | AvondN04 | 330342 | 304304 | 216218 | 165173 | 246252 | 352352 | | 317331 | 207217 | 137155 | 199227 | 353365 | 270280 | | 204206 | 177177 | 297303 |  |
| Coral Sea | Chesterfield | AvondN05 | 330338 | 304308 | 216218 | 173189 | 249261 | 354356 | | 325331 | 209215 | 149157 | 199207 | 347347 | 270270 | | 194200 | 175179 | 295301 |  |
| Coral Sea | Chesterfield | AvondN06 | 334350 | 300300 | 216216 | 165181 | 252255 | 352352 | | 321327 | 213243 | 153179 | 211223 | 351353 | 270280 | | 196200 | 151175 | 299301 |  |
| Coral Sea | Chesterfield | AvondN07 | 330346 | 304308 | 212216 | 165189 | 246252 | 352354 | | 311323 | 213243 | 163183 | 207227 | 347353 | 270280 | | 204214 | 175177 | 297301 |  |
| Coral Sea | Chesterfield | chesterfield01 | 334338 | 304312 | 216218 | 165187 | 246252 | 352354 | | 327333 | 207207 | 145147 | 207211 | 345353 | 270280 | | 194202 | 169173 | 301301 |  |
| Coral Sea | Chesterfield | chesterfield02 | 362362 | 304308 | 216218 | 165179 | 246252 | 352354 | | 327333 | 207207 | 145163 | 211211 | 345353 | 278278 | | 206214 | 169173 | 297301 |  |
| Coral Sea | Chesterfield | chesterfield03 | 334354 | 308312 | 216218 | 173209 | 252252 | 352352 | | 321333 | 207231 | 145147 | 199227 | 345361 | 272276 | | 194200 | 171175 | 301301 |  |
| Coral Sea | Chesterfield | chesterfield04 | 338354 | 304308 | 216220 | 179199 | 252252 | 352354 | | 319325 | 207213 | 161169 | 191199 | 345353 | 270280 | | 206206 | 155167 | 297301 |  |
| Coral Sea | Chesterfield | chesterfield05 | 334354 | 304308 | 216218 | 165189 | 252255 | 352354 | | 319327 | 207217 | 145151 | 211223 | 357357 | 266270 | | 206216 | 175175 | 293301 |  |
| Coral Sea | Chesterfield | chesterfield06 | 334354 | 300304 | 208218 | 173175 | 261261 | 352352 | | 319327 | 231245 | 145171 | 199223 | 345345 | 270278 | | 210214 | 173173 | 297301 |  |
| Coral Sea | Chesterfield | chesterfield07 | 342342 | 300300 | 216218 | 179203 | 249249 | 352352 | | 313327 | 229229 | 151151 | 191211 | 357357 | 268268 | | 206214 | 171171 | 287297 |  |
| Coral Sea | Chesterfield | chesterfield08 | 338362 | 304316 | 218218 | 165197 | 252252 | 352352 | | 319325 | 205207 | 161177 | 199227 | 345357 | 270280 | | 200206 | 173175 | 299301 |  |
| Coral Sea | Chesterfield | chesterfield09 | 326350 | 304308 | 212216 | 173187 | 252255 | 342352 | | 313327 | 207217 | 139151 | 211211 | 345353 | 266278 | | 202208 | 167175 | 287297 |  |
| Coral Sea | Chesterfield | chesterfield10 | 326350 | 304308 | 212216 | 173187 | 252255 | 352352 | | 309325 | 207217 | 139141 | 193211 | 345353 | 266278 | | 202208 | 171175 | 287297 |  |
| Coral Sea | Chesterfield | chesterfield11 | 342342 | 304308 | 212216 | 173187 | 252261 | 352352 | | 311311 | 207213 | 141151 | 193211 | 345353 | 266270 | | 202208 | 175175 | 287297 |  |
| Coral Sea | Chesterfield | chesterfield12 | 342342 | 308308 | 216216 | 193193 | 252261 | 354354 | | 313327 | 207217 | 163167 | 195223 | 000000 | 270270 | | 206206 | 173173 | 287297 |  |
| Coral Sea | Chesterfield | Renard01 | 350354 | 308312 | 216218 | 165189 | 249252 | 352352 | | 325327 | 223241 | 167175 | 191191 | 345345 | 270280 | | 202206 | 171173 | 301303 |  |
| Coral Sea | Chesterfield | Passage_ilot01 | 338358 | 292312 | 216218 | 173175 | 252258 | 352352 | | 315319 | 217243 | 169197 | 211223 | 349361 | 270272 | | 204206 | 171171 | 297303 |  |
| Coral Sea | Chesterfield | Passage01 | 330334 | 300304 | 216218 | 165185 | 246258 | 352352 | | 311329 | 243257 | 157177 | 199211 | 345357 | 266276 | | 206206 | 171177 | 297301 |  |
| Coral Sea | New Caledonia | Poindimie01 | 330358 | 304308 | 216218 | 169201 | 258264 | 352352 | | 331335 | 207221 | 167185 | 191211 | 345353 | 270270 | | 206206 | 169177 | 295303 |  |
| Coral Sea | New Caledonia | Poindimie02 | 350354 | 300300 | 216218 | 189191 | 240264 | 352352 | | 323331 | 217229 | 151175 | 191199 | 353353 | 270280 | | 194218 | 171175 | 293299 |  |
| Coral Sea | New Caledonia | Poindimie03 | 334346 | 304312 | 218220 | 165189 | 234249 | 352352 | | 323327 | 223243 | 161175 | 207227 | 351353 | 270270 | | 202216 | 173177 | 269301 |  |
| Coral Sea | New Caledonia | Poindimie04 | 322354 | 300308 | 216220 | 165169 | 252270 | 352352 | | 311319 | 207245 | 151169 | 211223 | 353357 | 270280 | | 194202 | 171173 | 269303 |  |
| Coral Sea | New Caledonia | Poindimie05 | 326338 | 304308 | 216220 | 179199 | 252252 | 352354 | | 319325 | 207213 | 161163 | 191199 | 345357 | 270280 | | 206206 | 155167 | 297301 |  |
| Coral Sea | New Caledonia | Belep01 | 318330 | 304316 | 216218 | 171173 | 246258 | 352352 | | 311321 | 207207 | 171179 | 191211 | 345359 | 270278 | | 200218 | 167175 | 297301 |  |
| Coral Sea | New Caledonia | Belep02 | 330350 | 304312 | 216218 | 165187 | 240258 | 352354 | | 311327 | 233259 | 159175 | 199211 | 355367 | 278278 | | 206216 | 169177 | 301301 |  |
| Coral Sea | New Caledonia | Belep03 | 338342 | 292312 | 214216 | 165171 | 252261 | 352352 | | 311319 | 213231 | 151181 | 199207 | 351353 | 270278 | | 206208 | 171175 | 297297 |  |
| Coral Sea | New Caledonia | Belep04 | 354370 | 308308 | 216218 | 185187 | 258261 | 354354 | | 321323 | 207231 | 157157 | 191223 | 353353 | 270278 | | 206206 | 151175 | 301301 |  |
| Coral Sea | New Caledonia | Belep05 | 350354 | 304304 | 216218 | 173175 | 252255 | 352352 | | 319333 | 213233 | 153177 | 211223 | 345357 | 270280 | | 204204 | 151175 | 297305 |  |
| Coral Sea | New Caledonia | Belep06 | 322334 | 308308 | 216218 | 189191 | 246252 | 352352 | | 311327 | 207207 | 151163 | 191211 | 345353 | 272280 | | 208212 | 171175 | 293301 |  |
| Coral Sea | New Caledonia | Belep07 | 330350 | 304316 | 216216 | 165173 | 246255 | 352352 | | 321329 | 211249 | 171189 | 199227 | 345355 | 272278 | | 208216 | 169171 | 301303 |  |
| Coral Sea | New Caledonia | Belep08 | 354358 | 304304 | 218220 | 197201 | 252252 | 352352 | | 317319 | 207219 | 149181 | 211223 | 353367 | 266278 | | 206206 | 167171 | 297301 |  |
| Mozambique Channel | Juan de Nova | Juan01 | 334374 | 300304 | 218218 | 169185 | 246261 | 346346 | | 327327 | 219221 | 159185 | 219223 | 361370 | 276278 | | 198198 | 165169 | 301301 |  |
| Mozambique Channel | Juan de Nova | Juan02 | 350358 | 300316 | 212218 | 169179 | 258261 | 346354 | | 325329 | 223255 | 147189 | 207235 | 351369 | 278278 | | 198198 | 167167 | 269301 |  |
| Mozambique Channel | Juan de Nova | Juan03 | 354354 | 300300 | 214216 | 169171 | 246258 | 346346 | | 327327 | 221221 | 141147 | 219223 | 351373 | 278278 | | 210210 | 167173 | 301301 |  |
| Mozambique Channel | Juan de Nova | Juan04 | 346362 | 304312 | 216216 | 169179 | 258258 | 346354 | | 327327 | 221245 | 141141 | 207219 | 367379 | 276278 | | 198210 | 167169 | 295301 |  |
| Mozambique Channel | Juan de Nova | Juan05 | 330370 | 300308 | 216216 | 169179 | 258258 | 346354 | | 327327 | 235235 | 141181 | 219219 | 349351 | 272276 | | 198206 | 169171 | 301301 |  |
| Mozambique Channel | Juan de Nova | Juan06 | 358362 | 300304 | 214218 | 179185 | 258264 | 346346 | | 339339 | 211221 | 159189 | 219235 | 351359 | 276278 | | 210220 | 171173 | 301303 |  |
| Mozambique Channel | Juan de Nova | Juan07 | 342358 | 300312 | 216218 | 169179 | 258264 | 346346 | | 327327 | 211225 | 143177 | 207219 | 351373 | 276280 | | 198214 | 165167 | 269269 |  |
| Mozambique Channel | Juan de Nova | Juan08 | 334334 | 304304 | 214216 | 179179 | 246261 | 346346 | | 329329 | 213223 | 141173 | 219235 | 369373 | 278280 | | 208210 | 167173 | 269301 |  |
| Mozambique Channel | Juan de Nova | Juan09 | 366366 | 300304 | 212212 | 169171 | 258264 | 346354 | | 327327 | 221259 | 143151 | 219219 | 349351 | 276278 | | 210214 | 167173 | 269301 |  |
| Mozambique Channel | Juan de Nova | Juan10 | 358358 | 304312 | 212218 | 179181 | 258264 | 346352 | | 327327 | 215249 | 149187 | 223223 | 351359 | 276276 | | 214216 | 167167 | 301305 |  |
| Mozambique Channel | Juan de Nova | Juan11 | 350362 | 300304 | 212212 | 169169 | 246252 | 346354 | | 329329 | 229229 | 151177 | 207223 | 349381 | 276276 | | 210210 | 169173 | 269269 |  |
| Mozambique Channel | Juan de Nova | Juan12 | 342354 | 300312 | 216216 | 169169 | 246261 | 346354 | | 327329 | 213249 | 141143 | 207207 | 349369 | 276276 | | 208210 | 167177 | 269301 |  |
| Mozambique Channel | Juan de Nova | Juan13 | 334338 | 304304 | 212216 | 169179 | 252258 | 346352 | | 327327 | 211221 | 141151 | 199199 | 351379 | 276278 | | 198210 | 167173 | 269301 |  |
| Mozambique Channel | Juan de Nova | Juan14 | 334334 | 304304 | 216216 | 179179 | 252264 | 346354 | | 327329 | 221247 | 143151 | 219223 | 359379 | 276278 | | 198214 | 169173 | 269301 |  |
| Mozambique Channel | Juan de Nova | Juan15 | 366366 | 300312 | 212216 | 169185 | 258264 | 346346 | | 327329 | 221223 | 175179 | 199199 | 351379 | 276280 | | 216216 | 169173 | 295303 |  |
| Mozambique Channel | Juan de Nova | Juan16 | 334350 | 300300 | 212218 | 179179 | 261264 | 346352 | | 327327 | 211221 | 141151 | 191199 | 345353 | 270278 | | 198198 | 167173 | 269301 |  |
| Mozambique Channel | Juan de Nova | Juan17 | 346354 | 300304 | 216218 | 179179 | 258264 | 346354 | | 327327 | 211223 | 163191 | 207219 | 371373 | 272278 | | 214216 | 169173 | 269303 |  |
| Mozambique Channel | Juan de Nova | Juan18 | 338358 | 304312 | 212218 | 179179 | 252258 | 346352 | | 327327 | 211215 | 147149 | 191223 | 349351 | 272276 | | 214214 | 167173 | 277301 |  |
| Mozambique Channel | Juan de Nova | Juan19 | 362370 | 300308 | 216218 | 177185 | 258264 | 346354 | | 329329 | 201221 | 141169 | 219219 | 351351 | 272276 | | 198210 | 169169 | 269269 |  |
| Mozambique Channel | Juan de Nova | Juan20 | 342358 | 300308 | 216218 | 179179 | 258258 | 346354 | | 323327 | 221251 | 143185 | 219235 | 349349 | 278278 | | 198216 | 173173 | 301301 |  |
| Mozambique Channel | Juan de Nova | Juan21 | 338362 | 304308 | 212212 | 179179 | 252258 | 346346 | | 327327 | 223225 | 151151 | 223235 | 351351 | 276278 | | 198218 | 167167 | 269269 |  |
| Mozambique Channel | Juan de Nova | Juan22 | 334346 | 300304 | 214216 | 179179 | 252264 | 346354 | | 325329 | 217221 | 143151 | 219223 | 359379 | 276278 | | 198214 | 173177 | 269301 |  |
| Mozambique Channel | Juan de Nova | JuanDagsit01 | 346354 | 300300 | 212220 | 169179 | 258258 | 346354 | | 327327 | 215221 | 141171 | 207219 | 375379 | 278280 | | 198210 | 169171 | 269305 |  |
| Mozambique Channel | Juan de Nova | JuanDagsit02 | 358358 | 300300 | 216218 | 179185 | 246252 | 346346 | | 327327 | 211221 | 175175 | 199207 | 351351 | 278278 | | 210214 | 171173 | 269305 |  |
| Mozambique Channel | Juan de Nova | JuanDagsit03 | 362362 | 300304 | 212218 | 169179 | 261264 | 346354 | | 327329 | 219221 | 151185 | 219235 | 349351 | 276276 | | 198210 | 167173 | 269295 |  |
| Mozambique Channel | Juan de Nova | JuanDagsit04 | 338362 | 300304 | 214218 | 179179 | 258258 | 346354 | | 329339 | 225251 | 141143 | 219235 | 349351 | 278280 | | 198198 | 171173 | 269301 |  |
| Mozambique Channel | Zelée Bank | Zelee01 | 366366 | 300304 | 212216 | 179179 | 246246 | 346346 | | 327327 | 225249 | 185185 | 207235 | 389399 | 278278 | | 198200 | 171173 | 301301 |  |
| Mozambique Channel | Zelée Bank | Zelee02 | 342366 | 300312 | 216216 | 169185 | 246264 | 346346 | | 327329 | 225233 | 143173 | 219219 | 371375 | 278278 | | 210216 | 169171 | 269301 |  |
| Mozambique Channel | Zelée Bank | Zelee03 | 330354 | 300300 | 214216 | 179179 | 258261 | 346346 | | 327329 | 217227 | 183191 | 207219 | 351357 | 276276 | | 198210 | 167171 | 269305 |  |
| Mozambique Channel | Zelée Bank | Zelee04 | 334358 | 300304 | 216218 | 169179 | 264264 | 346354 | | 327339 | 221225 | 141151 | 199235 | 351351 | 272278 | | 210214 | 169171 | 301301 |  |
| Mozambique Channel | Zelée Bank | Zelee05 | 366374 | 300312 | 216216 | 169183 | 252264 | 346354 | | 325327 | 221221 | 143151 | 219223 | 349351 | 276278 | | 198210 | 167169 | 301301 |  |
| Mozambique Channel | Zelée Bank | Zelee06 | 342342 | 300304 | 216218 | 169185 | 258258 | 346346 | | 327327 | 233243 | 151201 | 219235 | 349351 | 276276 | | 198198 | 167169 | 269305 |  |
| Mozambique Channel | Zelée Bank | Zelee07 | 330350 | 300304 | 214216 | 169179 | 252261 | 346346 | | 327343 | 215221 | 147177 | 219219 | 351369 | 276278 | | 198208 | 167173 | 299301 |  |
| North Central Pacific | Palmyra | Palmyra001 | 338342 | 304304 | 218218 | 183187 | 252252 | 352352 | | 321321 | 257259 | 155175 | 191211 | 347353 | 270278 | | 206206 | 173175 | 301303 |  |
| North Central Pacific | Palmyra | Palmyra002 | 338342 | 304308 | 216218 | 187205 | 246252 | 352354 | | 319321 | 207209 | 179179 | 191211 | 345363 | 270278 | | 206212 | 169173 | 295299 |  |
| North Central Pacific | Palmyra | Palmyra003 | 350350 | 312312 | 218220 | 167193 | 249252 | 352354 | | 327335 | 217225 | 159179 | 199211 | 345353 | 274276 | | 206206 | 173175 | 299301 |  |
| North Central Pacific | Palmyra | Palmyra004 | 334346 | 300304 | 216218 | 165171 | 252252 | 352354 | | 327331 | 205239 | 147173 | 223235 | 345345 | 270272 | | 200206 | 155175 | 295301 |  |
| North Central Pacific | Palmyra | Palmyra005 | 342350 | 300304 | 218218 | 165171 | 246252 | 352354 | | 331331 | 235243 | 173191 | 191223 | 353361 | 270280 | | 200206 | 169173 | 291295 |  |
| North Central Pacific | Palmyra | Palmyra006 | 342350 | 304312 | 216218 | 165171 | 252255 | 352352 | | 335349 | 217235 | 159189 | 211235 | 345353 | 270272 | | 206210 | 169173 | 299301 |  |
| North Central Pacific | Palmyra | Palmyra007 | 342350 | 296312 | 218218 | 171199 | 252270 | 352352 | | 321327 | 237253 | 147151 | 211235 | 353353 | 270280 | | 202208 | 169173 | 301303 |  |
| North Central Pacific | Palmyra | Palmyra008 | 318334 | 300300 | 216218 | 165171 | 249252 | 352352 | | 313331 | 223235 | 177189 | 207211 | 345359 | 270272 | | 200206 | 173175 | 301303 |  |
| North Central Pacific | Palmyra | Palmyra009 | 346354 | 304312 | 218218 | 187187 | 255261 | 352352 | | 313313 | 215215 | 147161 | 211235 | 345353 | 278282 | | 194200 | 173173 | 299301 |  |
| North Central Pacific | Palmyra | Palmyra010 | 334354 | 300304 | 218218 | 183201 | 252261 | 352352 | | 327327 | 247247 | 147153 | 199223 | 353357 | 270278 | | 206214 | 169173 | 291301 |  |
| North Central Pacific | Palmyra | Palmyra011 | 350350 | 300304 | 216218 | 183207 | 240252 | 352352 | | 319331 | 225225 | 161175 | 191223 | 353361 | 278282 | | 206206 | 169175 | 301303 |  |
| North Central Pacific | Palmyra | Palmyra012 | 346350 | 304312 | 218218 | 187193 | 249255 | 352354 | | 321327 | 217219 | 197199 | 191207 | 349353 | 278280 | | 202204 | 169173 | 297303 |  |
| North Central Pacific | Palmyra | Palmyra013 | 346350 | 308312 | 218218 | 171193 | 249252 | 352356 | | 329335 | 209241 | 157165 | 199223 | 345345 | 270278 | | 206206 | 171171 | 301301 |  |
| North Central Pacific | Palmyra | Palmyra014 | 334334 | 300312 | 218218 | 171189 | 255255 | 352354 | | 321329 | 225225 | 157165 | 207235 | 353355 | 278280 | | 206212 | 167179 | 297307 |  |
| North Central Pacific | Palmyra | Palmyra015 | 334342 | 300304 | 216218 | 165185 | 252252 | 352352 | | 327335 | 239243 | 161177 | 191211 | 359359 | 270276 | | 202206 | 175179 | 299303 |  |
| North Central Pacific | Palmyra | Palmyra016 | 350358 | 300312 | 216218 | 171173 | 249264 | 352352 | | 319331 | 227265 | 161165 | 211223 | 351353 | 270272 | | 206206 | 169173 | 301303 |  |
| North Central Pacific | Palmyra | Palmyra017 | 342342 | 304304 | 216218 | 193197 | 252261 | 352354 | | 313331 | 229229 | 151151 | 199207 | 345353 | 270278 | | 206208 | 171175 | 297299 |  |
| North Central Pacific | Palmyra | Palmyra018 | 326362 | 304312 | 216218 | 165165 | 255255 | 352352 | | 313319 | 233239 | 159171 | 199211 | 345345 | 270272 | | 206206 | 173173 | 293301 |  |
| North Central Pacific | Palmyra | Palmyra019 | 330342 | 308308 | 218218 | 183197 | 252264 | 352352 | | 327349 | 209267 | 151179 | 191207 | 353359 | 270270 | | 204206 | 169169 | 297325 |  |
| North Central Pacific | Palmyra | Palmyra020 | 334342 | 304304 | 218218 | 171187 | 252264 | 352352 | | 321321 | 217255 | 155155 | 191211 | 345353 | 270278 | | 202206 | 173175 | 297303 |  |
| North Central Pacific | Palmyra | Palmyra021 | 330346 | 304308 | 216220 | 171183 | 252252 | 352352 | | 321329 | 233265 | 155181 | 199211 | 345359 | 270278 | | 202206 | 171177 | 297301 |  |
| North Central Pacific | Palmyra | Palmyra022 | 326342 | 300308 | 216218 | 191197 | 255273 | 352352 | | 313319 | 243253 | 159183 | 223223 | 353353 | 270270 | | 200214 | 167171 | 295295 |  |
| North Central Pacific | Palmyra | Palmyra023 | 322334 | 292316 | 216218 | 201203 | 252252 | 352352 | | 313327 | 217227 | 181185 | 191223 | 345353 | 278280 | | 204206 | 167179 | 301301 |  |
| North Central Pacific | Palmyra | Palmyra024 | 338366 | 300304 | 218218 | 183183 | 252264 | 352352 | | 313327 | 217265 | 163165 | 191227 | 343353 | 270278 | | 206214 | 173175 | 299325 |  |
| North Central Pacific | Palmyra | Palmyra025 | 000000 | 304308 | 214214 | 171187 | 252252 | 354354 | | 313327 | 245245 | 145147 | 191191 | 345355 | 270272 | | 200208 | 173173 | 327339 |  |
| North Central Pacific | Palmyra | Palmyra026 | 322334 | 304308 | 218218 | 171201 | 252252 | 352352 | | 327331 | 235235 | 147185 | 191211 | 349353 | 272278 | | 202206 | 175175 | 327327 |  |
| North Central Pacific | Palmyra | Palmyra027 | 342350 | 308312 | 218218 | 189195 | 264264 | 352352 | | 327329 | 247265 | 151185 | 191199 | 345357 | 270270 | | 202206 | 175177 | 301303 |  |
| North Central Pacific | Palmyra | Palmyra028 | 338358 | 304304 | 218218 | 183189 | 252252 | 354354 | | 319329 | 207235 | 173189 | 211235 | 353357 | 266272 | | 206208 | 173175 | 293303 |  |
| North Central Pacific | Palmyra | Palmyra029 | 322342 | 304304 | 218218 | 171183 | 246252 | 352352 | | 327327 | 231249 | 167195 | 207235 | 343345 | 270278 | | 206206 | 171179 | 295325 |  |
| North Central Pacific | Palmyra | Palmyra030 | 318354 | 304304 | 216218 | 171193 | 246261 | 352352 | | 329331 | 209235 | 183185 | 211223 | 345353 | 270274 | | 192192 | 173175 | 301303 |  |
| North Central Pacific | Palmyra | Palmyra031 | 318342 | 304312 | 218218 | 183185 | 249261 | 352352 | | 319341 | 207271 | 159183 | 227227 | 353353 | 272278 | | 200204 | 171173 | 303305 |  |
| North Central Pacific | Palmyra | Palmyra032 | 330358 | 304308 | 218218 | 173183 | 261264 | 352352 | | 327331 | 217225 | 159197 | 191227 | 345369 | 270270 | | 206206 | 173175 | 301325 |  |
| North Central Pacific | Palmyra | Palmyra033 | 330346 | 304312 | 216218 | 185193 | 246252 | 352352 | | 313329 | 247259 | 151153 | 191191 | 345353 | 266278 | | 206206 | 175175 | 297301 |  |
| North Central Pacific | Palmyra | Palmyra034 | 334342 | 304308 | 216218 | 171183 | 252252 | 354354 | | 325335 | 207265 | 177177 | 211211 | 345345 | 270270 | | 210214 | 167169 | 297301 |  |
| North Central Pacific | Palmyra | Palmyra035 | 338342 | 308316 | 216218 | 189203 | 252255 | 352354 | | 327329 | 219225 | 163181 | 199207 | 353355 | 272278 | | 206208 | 171173 | 301303 |  |
| North Central Pacific | Palmyra | Palmyra036 | 330330 | 304304 | 218218 | 165165 | 261261 | 352352 | | 319327 | 243255 | 165191 | 191207 | 353357 | 268278 | | 212214 | 173173 | 301303 |  |
| North Central Pacific | Palmyra | Palmyra037 | 334366 | 304308 | 218218 | 197201 | 252252 | 352352 | | 329331 | 235235 | 153161 | 211223 | 343355 | 270270 | | 202206 | 171175 | 303303 |  |
| North Central Pacific | Palmyra | Palmyra038 | 342346 | 308312 | 218218 | 187201 | 249249 | 352352 | | 319321 | 207231 | 183199 | 211223 | 351353 | 278280 | | 206208 | 173177 | 303327 |  |
| North Central Pacific | Palmyra | Palmyra039 | 346350 | 296304 | 218218 | 185199 | 246252 | 352354 | | 323329 | 221225 | 155175 | 223223 | 353367 | 278278 | | 204212 | 173175 | 291301 |  |
| North Central Pacific | Palmyra | Palmyra040 | 330354 | 308308 | 216218 | 183183 | 246249 | 352354 | | 331357 | 219265 | 151167 | 207211 | 343353 | 272276 | | 208208 | 171173 | 295295 |  |
| North Central Pacific | Palmyra | Palmyra041 | 334366 | 304308 | 218218 | 197201 | 252252 | 352352 | | 329331 | 235235 | 153161 | 211223 | 343355 | 270270 | | 202206 | 171175 | 303303 |  |
| North Central Pacific | Palmyra | Palmyra042 | 330350 | 300300 | 218218 | 173193 | 252255 | 352354 | | 323349 | 237239 | 179183 | 211211 | 349353 | 270270 | | 202206 | 169173 | 301303 |  |
| North Central Pacific | Palmyra | Palmyra043 | 342354 | 312312 | 216218 | 171171 | 252264 | 352352 | | 327329 | 225237 | 147163 | 191223 | 345353 | 270278 | | 194206 | 167171 | 301301 |  |
| North Central Pacific | Palmyra | Palmyra044 | 330346 | 308312 | 216218 | 191207 | 252261 | 352352 | | 327333 | 207255 | 177185 | 211227 | 345361 | 270280 | | 204206 | 173175 | 291301 |  |
| North Central Pacific | Palmyra | Palmyra045 | 322354 | 300304 | 218218 | 171191 | 246252 | 352356 | | 319327 | 237253 | 153173 | 211227 | 353353 | 270276 | | 206206 | 175175 | 293297 |  |
| North Central Pacific | Palmyra | Palmyra046 | 338342 | 308312 | 218218 | 171191 | 246261 | 352354 | | 319329 | 227265 | 165173 | 191223 | 345353 | 270278 | | 206210 | 175179 | 303325 |  |
| North Central Pacific | Palmyra | Palmyra047 | 338342 | 296312 | 218218 | 185187 | 252258 | 352354 | | 331333 | 217259 | 153181 | 191211 | 345361 | 270278 | | 192206 | 169179 | 291325 |  |
| North Central Pacific | Palmyra | Palmyra048 | 326346 | 296304 | 218218 | 197205 | 252261 | 352352 | | 313319 | 219233 | 153169 | 203211 | 353355 | 270278 | | 206206 | 171175 | 299301 |  |
| North Central Pacific | Palmyra | Palmyra049 | 358366 | 304312 | 218218 | 165165 | 246246 | 352352 | | 335335 | 227249 | 163179 | 211227 | 353353 | 278278 | | 206206 | 171173 | 301301 |  |
| North Central Pacific | Palmyra | Palmyra050 | 342358 | 308316 | 218218 | 171181 | 240252 | 352354 | | 327333 | 259263 | 159165 | 191227 | 343367 | 270278 | | 204210 | 173179 | 307325 |  |
| North Central Pacific | Phoenix | Birnie604 | 326358 | 304308 | 216220 | 171197 | 255255 | 352354 | | 317327 | 233239 | 167175 | 211211 | 345359 | 270270 | | 212214 | 169175 | 299335 |  |
| North Central Pacific | Phoenix | Birnie605 | 326346 | 300312 | 218218 | 185191 | 255255 | 352356 | | 315327 | 207237 | 147167 | 227227 | 347357 | 270278 | | 212212 | 167173 | 297297 |  |
| North Central Pacific | Phoenix | Birnie606 | 342342 | 300304 | 218218 | 171171 | 252252 | 352352 | | 323327 | 219267 | 179185 | 211211 | 353353 | 276278 | | 192208 | 167173 | 295295 |  |
| North Central Pacific | Phoenix | Birnie607 | 346346 | 304308 | 216218 | 173183 | 252252 | 352354 | | 313327 | 221229 | 173189 | 207211 | 353371 | 272278 | | 206214 | 167173 | 297305 |  |
| North Central Pacific | Phoenix | Birnie608 | 358358 | 304304 | 218218 | 171187 | 252261 | 352352 | | 311319 | 215241 | 151157 | 199207 | 345345 | 278278 | | 208212 | 173173 | 301303 |  |
| North Central Pacific | Phoenix | Birnie609 | 346354 | 304312 | 218218 | 171193 | 252252 | 352352 | | 327343 | 217237 | 155173 | 191235 | 353355 | 270272 | | 200206 | 175175 | 301301 |  |
| North Central Pacific | Phoenix | Birnie610 | 342354 | 304308 | 216218 | 165171 | 252252 | 352354 | | 327333 | 235259 | 171171 | 199235 | 353369 | 280280 | | 206214 | 169169 | 301337 |  |
| North Central Pacific | Phoenix | Birnie611 | 358362 | 304308 | 218218 | 183185 | 246252 | 352352 | | 319329 | 207225 | 155167 | 211211 | 345353 | 272278 | | 200206 | 171175 | 301307 |  |
| North Central Pacific | Phoenix | Birnie612 | 338354 | 300304 | 216218 | 183193 | 252252 | 352352 | | 311327 | 207223 | 147191 | 211211 | 345353 | 270270 | | 206212 | 167173 | 301303 |  |
| North Central Pacific | Phoenix | Birnie613 | 326330 | 304308 | 216218 | 171193 | 246255 | 354354 | | 311331 | 207227 | 153177 | 211223 | 345353 | 270278 | | 204210 | 173177 | 293293 |  |
| North Central Pacific | Phoenix | Birnie614 | 330342 | 292296 | 218218 | 173173 | 246252 | 354354 | | 331335 | 237247 | 175181 | 191223 | 349349 | 270272 | | 204214 | 173175 | 299299 |  |
| North Central Pacific | Phoenix | Birnie615 | 318338 | 300308 | 218218 | 183193 | 252264 | 352352 | | 319327 | 207217 | 159177 | 211227 | 349353 | 278278 | | 210212 | 175175 | 269307 |  |
| North Central Pacific | Phoenix | Birnie616 | 330354 | 308312 | 216218 | 173183 | 252255 | 352352 | | 319333 | 207217 | 155173 | 191227 | 375379 | 278278 | | 206206 | 173175 | 291293 |  |
| North Central Pacific | Phoenix | Birnie617 | 358362 | 300308 | 212218 | 171193 | 252252 | 352352 | | 321331 | 209245 | 171179 | 191223 | 351351 | 270270 | | 212216 | 171175 | 297347 |  |
| North Central Pacific | Phoenix | Birnie618 | 322342 | 308308 | 216216 | 169189 | 252258 | 352354 | | 317321 | 261271 | 173185 | 191223 | 357365 | 276278 | | 200204 | 175179 | 303303 |  |
| North Central Pacific | Phoenix | Birnie619 | 330350 | 308312 | 216218 | 171189 | 252264 | 352352 | | 319327 | 209259 | 147177 | 199211 | 349351 | 272280 | | 192206 | 151175 | 297305 |  |
| North Central Pacific | Phoenix | Birnie620 | 338342 | 300308 | 218218 | 173187 | 252258 | 352354 | | 311319 | 207207 | 175183 | 191211 | 349351 | 270280 | | 202208 | 167175 | 301303 |  |
| North Central Pacific | Phoenix | Enderbury001 | 342342 | 304304 | 218220 | 185187 | 255255 | 352352 | | 315327 | 217241 | 171187 | 191223 | 345347 | 266270 | | 202212 | 175175 | 297303 |  |
| North Central Pacific | Phoenix | Enderbury004 | 346346 | 304304 | 216218 | 173207 | 252252 | 352352 | | 319333 | 245247 | 149153 | 191211 | 353353 | 270272 | | 200206 | 173173 | 339339 |  |
| North Central Pacific | Phoenix | Enderbury005 | 338338 | 300308 | 216218 | 185197 | 240252 | 352352 | | 315327 | 207239 | 159163 | 191207 | 345353 | 278282 | | 206206 | 171173 | 299301 |  |
| North Central Pacific | Phoenix | Enderbury006 | 342342 | 304308 | 218218 | 165185 | 246252 | 352354 | | 327335 | 273273 | 169175 | 211219 | 347361 | 272272 | | 206218 | 173175 | 301301 |  |
| North Central Pacific | Phoenix | Enderbury007 | 326326 | 300304 | 218218 | 165189 | 255255 | 352352 | | 327329 | 211269 | 159171 | 191223 | 349353 | 270270 | | 200208 | 173173 | 301303 |  |
| North Central Pacific | Phoenix | Enderbury008 | 330362 | 304304 | 218218 | 173175 | 252252 | 352354 | | 327331 | 239261 | 159163 | 191207 | 353353 | 270278 | | 200206 | 173175 | 305337 |  |
| North Central Pacific | Phoenix | Enderbury009 | 354354 | 304304 | 216218 | 189193 | 246246 | 352352 | | 327327 | 249271 | 165197 | 191191 | 345353 | 278278 | | 206208 | 169175 | 299329 |  |
| North Central Pacific | Phoenix | Enderbury011 | 342358 | 304312 | 218218 | 169183 | 252252 | 352354 | | 327331 | 207219 | 165177 | 211223 | 353369 | 272272 | | 206210 | 167173 | 297299 |  |
| North Central Pacific | Phoenix | Enderbury536 | 338354 | 308316 | 218218 | 165201 | 249249 | 352354 | | 327327 | 209227 | 163179 | 211223 | 353353 | 278278 | | 208212 | 173175 | 297327 |  |
| North Central Pacific | Phoenix | Enderbury537 | 338338 | 304308 | 218218 | 165173 | 246252 | 352352 | | 331335 | 215219 | 163191 | 211227 | 345359 | 266272 | | 206212 | 169173 | 291301 |  |
| North Central Pacific | Phoenix | Enderbury538 | 330350 | 292308 | 218220 | 173183 | 249249 | 352354 | | 319321 | 229229 | 171189 | 223223 | 353357 | 270278 | | 200206 | 167177 | 297303 |  |
| North Central Pacific | Phoenix | Enderbury539 | 334342 | 304304 | 218218 | 173179 | 249264 | 352352 | | 313335 | 221265 | 165185 | 211223 | 345349 | 270270 | | 206206 | 173173 | 297301 |  |
| North Central Pacific | Phoenix | Enderbury540 | 322354 | 308308 | 216218 | 173193 | 249252 | 352354 | | 313327 | 231239 | 165167 | 191207 | 353353 | 270272 | | 192216 | 171175 | 293295 |  |
| North Central Pacific | Phoenix | Enderbury541 | 342358 | 304312 | 216216 | 165173 | 252258 | 352352 | | 325327 | 217225 | 147161 | 191223 | 345359 | 280282 | | 206212 | 167175 | 295305 |  |
| North Central Pacific | Phoenix | Enderbury542 | 350354 | 296320 | 218218 | 185193 | 252255 | 352354 | | 327327 | 239249 | 161175 | 207207 | 353353 | 270276 | | 212212 | 169173 | 299301 |  |
| North Central Pacific | Phoenix | Enderbury543 | 342342 | 300312 | 216216 | 165173 | 252264 | 352352 | | 325331 | 213215 | 161171 | 203223 | 347359 | 274280 | | 206212 | 167177 | 295299 |  |
| North Central Pacific | Phoenix | Enderbury585 | 342346 | 296316 | 218218 | 165165 | 246252 | 352352 | | 311335 | 207235 | 185187 | 227235 | 353353 | 270276 | | 206208 | 169179 | 295301 |  |
| North Central Pacific | Phoenix | Enderbury586 | 342350 | 300308 | 218218 | 175189 | 252252 | 352352 | | 313331 | 229233 | 159175 | 223223 | 345359 | 270278 | | 212214 | 173173 | 293297 |  |
| North Central Pacific | Phoenix | Enderbury601 | 350350 | 308312 | 218218 | 171175 | 252252 | 352354 | | 311331 | 217217 | 171179 | 191223 | 349353 | 272278 | | 194204 | 167169 | 297301 |  |
| North Central Pacific | Phoenix | Enderbury602 | 326350 | 312312 | 216218 | 185193 | 252252 | 352352 | | 317317 | 229249 | 165187 | 207211 | 353353 | 270280 | | 206208 | 167171 | 297301 |  |
| North Central Pacific | Phoenix | Enderbury603 | 334334 | 304308 | 218218 | 169173 | 252252 | 352352 | | 319349 | 205229 | 147181 | 211211 | 345353 | 270278 | | 210210 | 171175 | 295303 |  |
| North Central Pacific | Phoenix | Kanton042 | 314330 | 300304 | 212218 | 173183 | 255255 | 352352 | | 321335 | 249249 | 151163 | 207211 | 345353 | 278278 | | 206208 | 157175 | 291301 |  |
| North Central Pacific | Phoenix | Kanton043 | 330346 | 304308 | 216218 | 173193 | 255255 | 352352 | | 321331 | 221259 | 169177 | 211211 | 345353 | 270278 | | 204206 | 173173 | 301303 |  |
| North Central Pacific | Phoenix | Kanton073 | 342358 | 300300 | 218218 | 173173 | 252252 | 354354 | | 319327 | 219235 | 159169 | 223223 | 353353 | 278278 | | 200208 | 173175 | 301305 |  |
| North Central Pacific | Phoenix | Kanton075 | 342350 | 296300 | 218218 | 165183 | 252255 | 352352 | | 327329 | 221265 | 151163 | 191227 | 345353 | 266278 | | 212214 | 169175 | 293329 |  |
| North Central Pacific | Phoenix | Kanton094 | 346346 | 304304 | 218218 | 173183 | 252252 | 352352 | | 319327 | 249249 | 159181 | 199211 | 345359 | 270280 | | 206206 | 167175 | 301303 |  |
| North Central Pacific | Phoenix | Kanton096 | 330346 | 304308 | 218218 | 175185 | 252264 | 352352 | | 317327 | 247247 | 155187 | 211223 | 349355 | 270278 | | 200200 | 169173 | 301305 |  |
| North Central Pacific | Phoenix | Kanton512 | 338358 | 300308 | 216218 | 187193 | 246252 | 352352 | | 311327 | 207233 | 153179 | 191191 | 345347 | 270278 | | 208210 | 169173 | 271301 |  |
| North Central Pacific | Phoenix | Kanton514 | 342350 | 300300 | 216218 | 171195 | 246252 | 348352 | | 311311 | 207233 | 169169 | 191199 | 347353 | 270272 | | 206212 | 155173 | 293303 |  |
| North Central Pacific | Phoenix | Kanton515 | 334338 | 304312 | 216220 | 171171 | 252252 | 352352 | | 313321 | 223223 | 155167 | 191235 | 347359 | 276278 | | 208210 | 173175 | 301339 |  |
| North Central Pacific | Phoenix | Kanton516 | 338354 | 300304 | 216218 | 171189 | 246255 | 352352 | | 329329 | 233265 | 151179 | 199223 | 353353 | 270280 | | 200206 | 167175 | 269297 |  |
| North Central Pacific | Phoenix | Kanton517 | 338350 | 304304 | 218220 | 171185 | 246252 | 352352 | | 311319 | 207233 | 175177 | 191211 | 353359 | 270278 | | 204206 | 155167 | 301303 |  |
| North Central Pacific | Phoenix | Kanton518 | 346350 | 308316 | 216218 | 173193 | 246246 | 352354 | | 331335 | 209223 | 163173 | 211227 | 357367 | 270282 | | 200208 | 155175 | 271293 |  |
| North Central Pacific | Phoenix | Kanton519 | 330350 | 304308 | 216218 | 165171 | 252252 | 352352 | | 331335 | 207251 | 169175 | 223235 | 345359 | 270278 | | 202212 | 167169 | 301303 |  |
| North Central Pacific | Phoenix | Kanton521 | 322322 | 300304 | 216218 | 173173 | 252252 | 352354 | | 319331 | 207217 | 155157 | 211211 | 359367 | 278278 | | 206208 | 173175 | 291301 |  |
| North Central Pacific | Phoenix | Kanton522 | 334342 | 304308 | 218218 | 173185 | 252270 | 352354 | | 327329 | 227227 | 163185 | 191219 | 345351 | 270280 | | 212214 | 157171 | 301301 |  |
| North Central Pacific | Phoenix | Kanton523 | 334350 | 300300 | 216218 | 189191 | 252255 | 352352 | | 315329 | 247247 | 177195 | 227235 | 347353 | 266276 | | 200206 | 173173 | 293299 |  |
| North Central Pacific | Phoenix | Kanton524 | 322362 | 304308 | 216218 | 189191 | 252261 | 352354 | | 327333 | 223239 | 159159 | 199227 | 353353 | 270278 | | 212218 | 157173 | 301301 |  |
| North Central Pacific | Phoenix | Kanton525 | 322326 | 304304 | 218218 | 183191 | 246252 | 352352 | | 319327 | 203241 | 145167 | 199227 | 349359 | 270272 | | 210212 | 173175 | 299343 |  |
| North Central Pacific | Phoenix | Kanton526 | 338342 | 308312 | 216218 | 185187 | 249255 | 352352 | | 319335 | 213221 | 153177 | 211211 | 353359 | 270278 | | 200214 | 173175 | 301303 |  |
| North Central Pacific | Phoenix | Kanton527 | 334350 | 300304 | 218218 | 173183 | 246252 | 352352 | | 323335 | 227235 | 179189 | 223227 | 357367 | 270278 | | 202204 | 173173 | 297301 |  |
| North Central Pacific | Phoenix | Kanton528 | 338358 | 300316 | 218218 | 173189 | 252252 | 352352 | | 327327 | 207207 | 149173 | 211211 | 353353 | 278278 | | 194206 | 173175 | 301337 |  |
| North Central Pacific | Phoenix | Kanton529 | 342342 | 300308 | 216218 | 187189 | 252255 | 352352 | | 319319 | 231231 | 149159 | 191199 | 353357 | 270278 | | 204206 | 167175 | 301335 |  |
| North Central Pacific | Phoenix | Kanton530 | 338342 | 296308 | 218218 | 181197 | 252252 | 352352 | | 317329 | 207265 | 163173 | 223223 | 345353 | 270270 | | 206216 | 167175 | 293303 |  |
| North Central Pacific | Phoenix | Kanton531 | 322322 | 304316 | 218218 | 165173 | 252252 | 352352 | | 319335 | 229239 | 175175 | 207211 | 345353 | 278278 | | 194210 | 173173 | 297303 |  |
| North Central Pacific | Phoenix | Kanton532 | 342362 | 304304 | 216218 | 165183 | 252252 | 354354 | | 319337 | 207243 | 171175 | 211223 | 345353 | 270280 | | 206208 | 167173 | 297299 |  |
| North Central Pacific | Phoenix | Kanton533 | 322322 | 304316 | 218218 | 193193 | 246252 | 352354 | | 327331 | 249251 | 179181 | 199223 | 345367 | 270276 | | 206208 | 167175 | 295297 |  |
| North Central Pacific | Phoenix | Kanton534 | 338370 | 300304 | 216218 | 193201 | 246246 | 352352 | | 313331 | 251265 | 159185 | 223235 | 355367 | 270272 | | 206210 | 169173 | 303303 |  |
| North Central Pacific | Phoenix | Manra651 | 322358 | 300308 | 216216 | 183201 | 246264 | 352354 | | 317335 | 207217 | 167177 | 223227 | 353353 | 270270 | | 200202 | 173175 | 295303 |  |
| North Central Pacific | Phoenix | Manra652 | 346350 | 300312 | 216218 | 183189 | 252255 | 352354 | | 331333 | 223249 | 171175 | 211211 | 345349 | 270280 | | 208214 | 173175 | 293347 |  |
| North Central Pacific | Phoenix | McKean696 | 342350 | 304304 | 216218 | 183185 | 252252 | 352352 | | 315319 | 229229 | 157159 | 191211 | 355357 | 270278 | | 194206 | 169171 | 337339 |  |
| North Central Pacific | Phoenix | McKean697 | 330338 | 308308 | 216218 | 171171 | 252252 | 352352 | | 321331 | 225265 | 157163 | 211211 | 347349 | 278278 | | 192206 | 167169 | 291301 |  |
| North Central Pacific | Phoenix | McKean698 | 326338 | 304312 | 216216 | 177187 | 252255 | 352352 | | 319321 | 207253 | 155159 | 191191 | 353353 | 278278 | | 194204 | 175175 | 303303 |  |
| North Central Pacific | Phoenix | McKean699 | 346346 | 300308 | 218218 | 175191 | 255255 | 352354 | | 327327 | 207243 | 181189 | 191211 | 343367 | 270272 | | 206212 | 173177 | 297303 |  |
| North Central Pacific | Phoenix | McKean700 | 322322 | 296316 | 218218 | 173183 | 246264 | 352352 | | 327333 | 217225 | 151157 | 191235 | 353353 | 278278 | | 206212 | 169173 | 291293 |  |
| North Central Pacific | Phoenix | McKean701 | 338362 | 300308 | 218218 | 171189 | 246252 | 352352 | | 329333 | 207213 | 177191 | 223223 | 353367 | 270274 | | 192206 | 169175 | 303333 |  |
| North Central Pacific | Phoenix | McKean702 | 338350 | 308308 | 216218 | 165189 | 252252 | 352352 | | 319333 | 207213 | 159161 | 223223 | 349353 | 272272 | | 210212 | 171175 | 297301 |  |
| North Central Pacific | Phoenix | McKean703 | 334338 | 300304 | 218218 | 159165 | 240252 | 352352 | | 317329 | 247247 | 169169 | 191227 | 353353 | 278278 | | 206210 | 173175 | 303339 |  |
| North Central Pacific | Phoenix | McKean704 | 342354 | 300308 | 218218 | 171195 | 252261 | 352352 | | 315317 | 249265 | 165185 | 191211 | 345353 | 272276 | | 192206 | 171175 | 293303 |  |
| North Central Pacific | Phoenix | McKean705 | 330354 | 300304 | 218218 | 165185 | 246252 | 352352 | | 327331 | 223261 | 163171 | 207223 | 361369 | 270278 | | 192206 | 173177 | 293303 |  |
| North Central Pacific | Phoenix | McKean710 | 334354 | 304308 | 216218 | 183197 | 252252 | 352352 | | 327327 | 203267 | 155159 | 207223 | 345345 | 278278 | | 206212 | 175177 | 297301 |  |
| North Central Pacific | Phoenix | McKean714 | 346362 | 300304 | 212218 | 183191 | 252261 | 352352 | | 311333 | 211225 | 169179 | 199199 | 353361 | 270278 | | 200206 | 171179 | 299301 |  |
| North Central Pacific | Phoenix | McKean715 | 354354 | 304308 | 218218 | 165173 | 246252 | 352352 | | 327339 | 245265 | 171181 | 199207 | 343347 | 274278 | | 206206 | 171175 | 301303 |  |
| North Central Pacific | Phoenix | McKean716 | 322350 | 300304 | 216218 | 167183 | 246252 | 352352 | | 311321 | 207231 | 147151 | 191199 | 353357 | 270282 | | 206214 | 167169 | 295305 |  |
| North Central Pacific | Phoenix | McKean717 | 322346 | 296304 | 218218 | 173185 | 252252 | 352352 | | 321321 | 271271 | 147157 | 203227 | 353361 | 270272 | | 202212 | 167177 | 299301 |  |
| North Central Pacific | Phoenix | McKean718 | 342346 | 304308 | 216218 | 171171 | 252261 | 352352 | | 333333 | 205255 | 153171 | 211223 | 345345 | 274278 | | 206206 | 155173 | 297303 |  |
| North Central Pacific | Phoenix | McKean719 | 350350 | 296304 | 218218 | 165183 | 246252 | 352354 | | 313321 | 223223 | 147151 | 211235 | 345347 | 270278 | | 206206 | 169175 | 299313 |  |
| North Central Pacific | Phoenix | McKean720 | 322358 | 296304 | 216218 | 191193 | 252252 | 352354 | | 319331 | 231239 | 151163 | 191227 | 345345 | 276278 | | 200208 | 171175 | 293297 |  |
| North Central Pacific | Phoenix | McKean721 | 342342 | 296304 | 216218 | 173183 | 246252 | 352354 | | 329341 | 249249 | 153175 | 191223 | 353353 | 270270 | | 206206 | 173175 | 301303 |  |
| North Central Pacific | Phoenix | McKean722 | 342346 | 304304 | 218220 | 189191 | 252252 | 352354 | | 311319 | 223229 | 173183 | 203207 | 355355 | 274278 | | 206214 | 175175 | 303303 |  |
| North Central Pacific | Phoenix | McKean723 | 326338 | 308312 | 218218 | 173183 | 252252 | 352352 | | 313343 | 207265 | 159173 | 223227 | 345355 | 270278 | | 208214 | 173175 | 297299 |  |
| North Central Pacific | Phoenix | McKean724 | 338354 | 304304 | 216218 | 171183 | 252252 | 354354 | | 311315 | 235249 | 147165 | 203223 | 351365 | 266280 | | 212216 | 167175 | 301303 |  |
| North Central Pacific | Phoenix | McKean725 | 342350 | 308308 | 216218 | 171201 | 252252 | 352354 | | 315333 | 211265 | 147165 | 191191 | 345353 | 270272 | | 206206 | 171179 | 297303 |  |
| North Central Pacific | Phoenix | McKean726 | 342354 | 316316 | 216218 | 173191 | 246252 | 352352 | | 317347 | 207207 | 177191 | 223223 | 345355 | 272274 | | 200212 | 173175 | 301301 |  |
| North Central Pacific | Phoenix | Niku289 | 338342 | 304304 | 216218 | 173185 | 246252 | 352356 | | 317319 | 217217 | 159181 | 207223 | 345347 | 266278 | | 204204 | 173173 | 303329 |  |
| North Central Pacific | Phoenix | Niku291 | 334354 | 296304 | 218220 | 171189 | 252255 | 352352 | | 327335 | 205223 | 177191 | 207219 | 345353 | 270270 | | 208212 | 167173 | 297297 |  |
| North Central Pacific | Phoenix | Niku292 | 350350 | 296304 | 216218 | 173183 | 240252 | 352352 | | 321327 | 243247 | 161163 | 199223 | 357359 | 266280 | | 210210 | 169175 | 293301 |  |
| North Central Pacific | Phoenix | Niku293 | 342354 | 304308 | 218218 | 183191 | 252252 | 352352 | | 321331 | 205257 | 147171 | 191211 | 353353 | 270280 | | 200208 | 155175 | 291301 |  |
| North Central Pacific | Phoenix | Niku294 | 322350 | 308308 | 216218 | 193197 | 246246 | 352354 | | 321329 | 247247 | 155163 | 199227 | 353367 | 272276 | | 208214 | 169171 | 303339 |  |
| North Central Pacific | Phoenix | Niku295 | 338338 | 300312 | 212216 | 173175 | 252252 | 352352 | | 311321 | 227263 | 175183 | 219219 | 347353 | 278278 | | 208210 | 169175 | 297301 |  |
| North Central Pacific | Phoenix | Niku296 | 326374 | 304304 | 216216 | 173183 | 255255 | 352352 | | 313329 | 217227 | 147185 | 191223 | 345353 | 270270 | | 200212 | 169175 | 297297 |  |
| North Central Pacific | Phoenix | Niku297 | 314346 | 296312 | 216216 | 173193 | 264264 | 352356 | | 321333 | 207223 | 147155 | 191235 | 345345 | 272280 | | 194206 | 171177 | 297299 |  |
| North Central Pacific | Phoenix | Niku298 | 326334 | 296320 | 218218 | 173175 | 252252 | 352354 | | 321353 | 217241 | 171179 | 211223 | 353353 | 278280 | | 206206 | 155167 | 293297 |  |
| North Central Pacific | Phoenix | Niku299 | 334338 | 300300 | 218220 | 165183 | 252264 | 352354 | | 313319 | 265265 | 153175 | 207235 | 349365 | 270270 | | 200214 | 173175 | 295301 |  |
| North Central Pacific | Phoenix | Niku331 | 334342 | 308308 | 218218 | 165187 | 252252 | 352352 | | 317329 | 203245 | 159181 | 223235 | 349349 | 270270 | | 206206 | 155175 | 297301 |  |
| North Central Pacific | Phoenix | Niku334 | 350354 | 304316 | 218218 | 165183 | 252255 | 352352 | | 311319 | 233233 | 153175 | 199223 | 345353 | 270280 | | 200208 | 155175 | 297301 |  |
| North Central Pacific | Phoenix | Niku337 | 338370 | 300304 | 216218 | 165191 | 246252 | 352352 | | 325349 | 223223 | 159187 | 191223 | 345353 | 276278 | | 206208 | 173175 | 293297 |  |
| North Central Pacific | Phoenix | Niku355 | 330346 | 304304 | 216218 | 165183 | 252252 | 352352 | | 327327 | 223223 | 153159 | 227227 | 347357 | 278282 | | 206206 | 175175 | 299303 |  |
| North Central Pacific | Phoenix | Niku356 | 338374 | 304304 | 216218 | 183189 | 252255 | 352352 | | 329331 | 217251 | 147169 | 211223 | 345347 | 270272 | | 206212 | 155173 | 293303 |  |
| North Central Pacific | Phoenix | Niku357 | 338346 | 304312 | 218218 | 167183 | 252252 | 352352 | | 329333 | 207207 | 153171 | 215227 | 353357 | 280280 | | 210212 | 169173 | 301307 |  |
| North Central Pacific | Phoenix | Niku361 | 318342 | 304304 | 218220 | 171197 | 246252 | 352352 | | 319331 | 241251 | 159167 | 211223 | 347359 | 278280 | | 206206 | 173173 | 287299 |  |
| North Central Pacific | Phoenix | Niku362 | 322366 | 304304 | 218218 | 185193 | 252255 | 352354 | | 321327 | 247247 | 177183 | 235235 | 345357 | 270272 | | 194206 | 167175 | 299299 |  |
| North Central Pacific | Phoenix | Niku363 | 338338 | 304312 | 218218 | 173177 | 246252 | 352352 | | 319335 | 243265 | 159169 | 199211 | 353353 | 270278 | | 206212 | 173175 | 297301 |  |
| North Central Pacific | Phoenix | Niku365 | 326346 | 312312 | 218218 | 189197 | 252252 | 352352 | | 311327 | 217227 | 161177 | 191227 | 353357 | 270272 | | 210212 | 171175 | 297303 |  |
| North Central Pacific | Phoenix | Niku366 | 366366 | 304308 | 216216 | 183189 | 252252 | 352354 | | 313331 | 271271 | 179183 | 207207 | 345353 | 276278 | | 206206 | 173173 | 299339 |  |
| North Central Pacific | Phoenix | Niku369 | 338342 | 300304 | 218218 | 165173 | 264264 | 352352 | | 311327 | 231267 | 165195 | 199227 | 345353 | 270278 | | 210210 | 171173 | 297299 |  |
| North Central Pacific | Phoenix | Niku375 | 346358 | 300304 | 212218 | 187189 | 252252 | 352352 | | 327331 | 223265 | 151161 | 211211 | 351383 | 270270 | | 200204 | 173177 | 293297 |  |
| North Central Pacific | Phoenix | Niku381 | 334334 | 296316 | 218218 | 175189 | 252252 | 352352 | | 313319 | 233247 | 155185 | 223235 | 345353 | 270278 | | 206208 | 173179 | 301301 |  |
| North Central Pacific | Phoenix | Niku384 | 338346 | 304308 | 218218 | 183187 | 246252 | 352352 | | 319329 | 229269 | 159163 | 199223 | 347353 | 272278 | | 206206 | 169169 | 293303 |  |
| North Central Pacific | Phoenix | Niku385 | 334334 | 304308 | 218218 | 173189 | 252264 | 352352 | | 321323 | 225263 | 157179 | 191211 | 353357 | 270270 | | 206214 | 175175 | 301305 |  |
| North Central Pacific | Phoenix | Niku386 | 342342 | 296300 | 216218 | 173175 | 246255 | 352354 | | 321327 | 243265 | 169179 | 211211 | 345353 | 278278 | | 206212 | 167173 | 287295 |  |
| North Central Pacific | Phoenix | Niku387 | 334350 | 296304 | 218218 | 165175 | 246252 | 352354 | | 313335 | 207269 | 155177 | 207235 | 345353 | 270272 | | 200206 | 175175 | 299343 |  |
| North Central Pacific | Phoenix | Niku735 | 342350 | 300304 | 218218 | 173185 | 252252 | 352352 | | 313315 | 227233 | 171173 | 211211 | 353353 | 270270 | | 192210 | 173173 | 299301 |  |
| North Central Pacific | Phoenix | Niku737 | 334350 | 304308 | 216218 | 193199 | 252255 | 352354 | | 313317 | 231261 | 157177 | 199219 | 357367 | 270272 | | 206210 | 155167 | 303337 |  |
| North Central Pacific | Phoenix | Niku738 | 322354 | 300316 | 216218 | 183189 | 249252 | 352352 | | 319319 | 217233 | 147175 | 211227 | 345353 | 270278 | | 206210 | 167173 | 301303 |  |
| North Central Pacific | Phoenix | Niku739 | 338354 | 304316 | 218218 | 171185 | 246255 | 352352 | | 321327 | 225271 | 179185 | 211211 | 353361 | 270270 | | 204206 | 171175 | 301301 |  |
| North Central Pacific | Phoenix | Niku740 | 362362 | 300312 | 218218 | 183189 | 249252 | 352352 | | 327329 | 221245 | 153169 | 223227 | 353353 | 270274 | | 200206 | 169175 | 295301 |  |
| North Central Pacific | Phoenix | Niku741 | 342346 | 300304 | 218218 | 193197 | 252252 | 352352 | | 331343 | 207247 | 169171 | 207215 | 345367 | 270278 | | 200206 | 173175 | 291301 |  |
| North Central Pacific | Phoenix | Niku742 | 330334 | 300308 | 216218 | 171193 | 252252 | 352354 | | 329335 | 215247 | 171183 | 199211 | 347353 | 278280 | | 192208 | 169175 | 295303 |  |
| North Central Pacific | Phoenix | Niku743 | 334338 | 308312 | 216218 | 173193 | 252252 | 352352 | | 329331 | 207251 | 175177 | 199199 | 345365 | 278278 | | 192204 | 173173 | 301339 |  |
| North Central Pacific | Phoenix | Niku744 | 354354 | 300308 | 216218 | 183187 | 246252 | 352352 | | 327331 | 209245 | 153167 | 211223 | 345357 | 270278 | | 204210 | 175179 | 297339 |  |
| North Central Pacific | Phoenix | Niku745 | 338370 | 300304 | 216218 | 165191 | 246252 | 352352 | | 325347 | 223231 | 179189 | 191191 | 345353 | 276278 | | 206208 | 173175 | 293297 |  |
| North Central Pacific | Phoenix | Niku747 | 330338 | 300308 | 218218 | 171205 | 246252 | 352352 | | 319339 | 241265 | 175185 | 199211 | 359365 | 270280 | | 210212 | 155171 | 301339 |  |
| North Central Pacific | Phoenix | Niku762 | 334338 | 300300 | 218220 | 165183 | 252264 | 352354 | | 313319 | 265265 | 153175 | 207235 | 349365 | 270270 | | 200214 | 173175 | 295301 |  |
| North Central Pacific | Phoenix | Niku784 | 330334 | 304304 | 218218 | 165183 | 246252 | 352354 | | 325327 | 207247 | 175177 | 199227 | 353353 | 270272 | | 200206 | 169175 | 295297 |  |
| North Central Pacific | Phoenix | Niku805 | 318354 | 304316 | 218218 | 193197 | 246246 | 352352 | | 319319 | 235251 | 159167 | 223223 | 353359 | 280280 | | 204206 | 167173 | 287293 |  |
| North Central Pacific | Phoenix | Niku806 | 354354 | 304308 | 218218 | 173187 | 252252 | 352352 | | 323329 | 217217 | 155155 | 199211 | 345355 | 270270 | | 200206 | 173175 | 303327 |  |
| North Central Pacific | Phoenix | Niku816 | 334338 | 300304 | 218218 | 189197 | 252252 | 352352 | | 327329 | 237247 | 175175 | 191211 | 353353 | 278278 | | 200206 | 167177 | 303303 |  |
| North Central Pacific | Phoenix | Orona151 | 326346 | 300312 | 218218 | 173193 | 249252 | 352352 | | 329333 | 207231 | 163163 | 199223 | 345353 | 270274 | | 212212 | 169175 | 287305 |  |
| North Central Pacific | Phoenix | Orona184 | 334358 | 300304 | 212218 | 173183 | 246264 | 352354 | | 321323 | 207207 | 151187 | 211211 | 365365 | 276278 | | 204206 | 169175 | 279301 |  |
| North Central Pacific | Phoenix | Orona186 | 322358 | 300304 | 218218 | 173189 | 249255 | 352352 | | 319327 | 247247 | 175181 | 203211 | 345353 | 270272 | | 200206 | 175175 | 305307 |  |
| North Central Pacific | Phoenix | Orona192 | 350354 | 300304 | 216218 | 173175 | 255255 | 352356 | | 321327 | 219227 | 147161 | 191223 | 353359 | 270278 | | 208214 | 155173 | 287301 |  |
| North Central Pacific | Phoenix | Orona218 | 342366 | 300304 | 216218 | 165189 | 252252 | 352352 | | 321327 | 241241 | 163165 | 199223 | 345367 | 270280 | | 192200 | 173173 | 299303 |  |
| North Central Pacific | Phoenix | Orona219 | 314354 | 304304 | 216218 | 185189 | 252252 | 352352 | | 313327 | 227247 | 177189 | 191223 | 345353 | 270270 | | 206216 | 167173 | 295297 |  |
| North Central Pacific | Phoenix | Orona221 | 350350 | 304304 | 218218 | 173175 | 246252 | 352354 | | 327327 | 207245 | 185185 | 227227 | 345353 | 274278 | | 206212 | 169173 | 301303 |  |
| North Central Pacific | Phoenix | Orona223 | 342366 | 300304 | 216218 | 169173 | 246252 | 352352 | | 319349 | 223223 | 155159 | 191211 | 359359 | 270272 | | 204206 | 165173 | 301301 |  |
| North Central Pacific | Phoenix | Orona270 | 342346 | 296304 | 218218 | 173187 | 246261 | 352352 | | 319319 | 251251 | 157179 | 207227 | 345355 | 272278 | | 206206 | 175175 | 299299 |  |
| North Central Pacific | Phoenix | Orona653 | 326338 | 300304 | 214218 | 189193 | 252252 | 352352 | | 319327 | 265265 | 155155 | 191211 | 345345 | 270278 | | 204206 | 173173 | 293303 |  |
| North Central Pacific | Phoenix | Orona654 | 342354 | 304304 | 216218 | 185201 | 252252 | 352352 | | 319331 | 243269 | 167173 | 223227 | 349353 | 270276 | | 200200 | 173181 | 301307 |  |
| North Central Pacific | Phoenix | Orona679 | 350370 | 304312 | 218218 | 171183 | 246252 | 352352 | | 327335 | 205223 | 153157 | 191199 | 353359 | 270272 | | 200206 | 173175 | 301339 |  |
| North Central Pacific | Phoenix | Rawaki413 | 338338 | 296304 | 218218 | 179195 | 246252 | 352352 | | 323329 | 219265 | 175179 | 211211 | 345351 | 270278 | | 208214 | 169175 | 291295 |  |
| North Central Pacific | Phoenix | Rawaki414 | 318346 | 304312 | 216218 | 171193 | 240252 | 352354 | | 313331 | 207233 | 159163 | 211231 | 349355 | 266272 | | 200206 | 173173 | 293307 |  |
| North Central Pacific | Phoenix | Rawaki415 | 346354 | 304312 | 218218 | 165183 | 240252 | 352352 | | 325331 | 231247 | 159167 | 199199 | 345357 | 276278 | | 200206 | 169175 | 301339 |  |
| North Central Pacific | Phoenix | Rawaki644 | 342354 | 296300 | 216218 | 173183 | 252258 | 352352 | | 321335 | 207207 | 159159 | 211211 | 353367 | 278278 | | 210214 | 167173 | 301303 |  |
| North Central Pacific | Phoenix | Rawaki645 | 322358 | 304304 | 218218 | 181191 | 249252 | 352352 | | 327351 | 217243 | 167183 | 191199 | 353355 | 278280 | | 206206 | 151175 | 293301 |  |
| North Central Pacific | Phoenix | Rawaki646 | 338350 | 304304 | 218218 | 173195 | 264264 | 352354 | | 325325 | 235259 | 159173 | 211211 | 349361 | 278280 | | 200206 | 169175 | 301301 |  |
| North Central Pacific | Phoenix | Rawaki647 | 322322 | 308312 | 216218 | 165187 | 252252 | 352354 | | 323331 | 215227 | 171183 | 199223 | 347361 | 270270 | | 200206 | 169173 | 301301 |  |
| North Central Pacific | Phoenix | Rawaki648 | 322358 | 300304 | 216218 | 165209 | 252261 | 352354 | | 321325 | 217251 | 157175 | 199223 | 345353 | 270272 | | 206206 | 169175 | 301301 |  |
| North Central Pacific | Phoenix | Rawaki649 | 334354 | 304312 | 216216 | 173173 | 246261 | 352354 | | 319331 | 223261 | 171175 | 199211 | 353353 | 272280 | | 206206 | 175177 | 301301 |  |
| North Central Pacific | Phoenix | Rawaki650 | 322350 | 304304 | 216218 | 183201 | 246252 | 352352 | | 327335 | 207273 | 157183 | 203227 | 347347 | 266272 | | 200204 | 171175 | 291297 |  |
| North Central Pacific | Phoenix | Rawaki713 | 326342 | 300304 | 218218 | 181203 | 264264 | 352352 | | 329335 | 223253 | 173175 | 199207 | 347349 | 272280 | | 200208 | 173175 | 295301 |  |
| North Central Pacific | Phoenix | Winslow275 | 326346 | 304308 | 216216 | 183187 | 252255 | 352352 | | 319333 | 235241 | 153159 | 211211 | 349353 | 270270 | | 206210 | 167175 | 301353 |  |
| North Central Pacific | Phoenix | Winslow276 | 338338 | 304312 | 218218 | 193197 | 252261 | 352352 | | 321335 | 241249 | 153179 | 219223 | 353357 | 270270 | | 192208 | 173173 | 297301 |  |
| North Central Pacific | Phoenix | Winslow277 | 326342 | 304308 | 216218 | 185189 | 249258 | 352354 | | 321333 | 251251 | 157159 | 191207 | 345351 | 266278 | | 200206 | 155173 | 295301 |  |
| North Central Pacific | Phoenix | Winslow278 | 334342 | 296308 | 216216 | 183191 | 246252 | 352352 | | 319327 | 207239 | 151179 | 191223 | 345357 | 270270 | | 202208 | 171173 | 297301 |  |
| North Central Pacific | Phoenix | Winslow282 | 334342 | 304304 | 212218 | 165189 | 252252 | 352354 | | 317317 | 207247 | 155159 | 211227 | 353355 | 272276 | | 200204 | 171177 | 297305 |  |
| North Central Pacific | Phoenix | Winslow283 | 330338 | 304312 | 218218 | 175187 | 255264 | 352352 | | 325335 | 207263 | 155181 | 211235 | 353353 | 270278 | | 200210 | 175175 | 301303 |  |
| South Central Pacific | Society | Motu01 | 330358 | 304312 | 218218 | 175189 | 252255 | 352352 | | 311329 | 207265 | 183185 | 223223 | 345353 | 272278 | | 206208 | 169173 | 301337 |  |
| South Central Pacific | Society | Motu02 | 354354 | 300316 | 216218 | 171185 | 240255 | 352352 | | 311335 | 241243 | 177181 | 199223 | 357361 | 270270 | | 206206 | 169175 | 295299 |  |
| South Central Pacific | Society | Motu03 | 330350 | 308312 | 216218 | 187199 | 252252 | 354354 | | 311327 | 217229 | 153177 | 207223 | 353355 | 274280 | | 206206 | 169169 | 295303 |  |
| South Central Pacific | Society | Polynesie20 | 342362 | 304308 | 218218 | 169193 | 252252 | 352354 | | 331331 | 207229 | 153183 | 191227 | 345345 | 272276 | | 200206 | 173175 | 293301 |  |
| South Central Pacific | Society | Polynesie21 | 330354 | 300316 | 218218 | 171191 | 246255 | 352352 | | 319333 | 227245 | 173183 | 191207 | 345355 | 274280 | | 212214 | 169175 | 301303 |  |
| South Central Pacific | Society | Polynesie22 | 338350 | 308308 | 218218 | 171171 | 252252 | 352354 | | 311329 | 215239 | 147187 | 207211 | 349359 | 270272 | | 206210 | 169173 | 299303 |  |
| South Central Pacific | Society | Polynesie23 | 334350 | 304308 | 216218 | 181197 | 246252 | 352354 | | 311311 | 225237 | 147161 | 211223 | 345359 | 270270 | | 206212 | 173179 | 293293 |  |
| South Central Pacific | Society | Polynesie24 | 342346 | 300308 | 218218 | 189191 | 246252 | 352352 | | 327329 | 223227 | 173173 | 223223 | 345349 | 276278 | | 206214 | 167173 | 297303 |  |
| South Central Pacific | Society | Tiki01 | 318338 | 300300 | 218218 | 183201 | 252252 | 352352 | | 327331 | 213229 | 181181 | 191219 | 345353 | 270274 | | 210210 | 167167 | 297299 |  |
| South Central Pacific | Society | Tahiti38 | 330330 | 300308 | 218218 | 165173 | 252252 | 352352 | | 327329 | 235235 | 181185 | 219223 | 345345 | 270280 | | 204214 | 173179 | 301303 |  |
| South Central Pacific | Society | Tahiti39 | 326330 | 304304 | 216218 | 165189 | 252255 | 352352 | | 313331 | 215247 | 147173 | 207223 | 353353 | 270278 | | 206206 | 169175 | 301301 |  |
| South Central Pacific | Society | Tahiti40 | 338342 | 308312 | 216218 | 173187 | 246246 | 352352 | | 333335 | 209265 | 147183 | 199223 | 345357 | 270278 | | 206208 | 169173 | 333333 |  |
| South Central Pacific | Society | Tahiti41 | 350350 | 300304 | 218218 | 173175 | 249249 | 352354 | | 313327 | 207231 | 159169 | 199211 | 345345 | 270280 | | 200212 | 169173 | 293303 |  |
| South Central Pacific | Society | Tahiti42 | 326330 | 296304 | 218218 | 173173 | 252252 | 352352 | | 313327 | 233235 | 183183 | 207211 | 345355 | 280280 | | 206212 | 169175 | 301301 |  |
| South Central Pacific | Tuamotu | Tuam01 | 302326 | 304308 | 216218 | 187189 | 252255 | 352352 | | 313313 | 209227 | 165183 | 207207 | 345349 | 270278 | | 204212 | 169177 | 299299 |  |
| South Central Pacific | Tuamotu | Tuam02 | 302342 | 308312 | 218218 | 173187 | 246246 | 352352 | | 319327 | 235235 | 167169 | 207223 | 349353 | 270278 | | 204210 | 171173 | 301303 |  |
| South Central Pacific | Tuamotu | Tuam03 | 338342 | 296308 | 218218 | 187201 | 246246 | 352354 | | 313327 | 215233 | 147161 | 211223 | 345357 | 270278 | | 206212 | 169173 | 293301 |  |
| South Central Pacific | Tuamotu | Tuam04 | 338342 | 296308 | 218218 | 187201 | 246246 | 352354 | | 311327 | 215233 | 147161 | 211223 | 345357 | 270278 | | 206212 | 169173 | 293301 |  |
| South Central Pacific | Tuamotu | Tuam05 | 334350 | 300304 | 216218 | 183191 | 246261 | 352352 | | 311327 | 239239 | 153181 | 191223 | 347353 | 270272 | | 206208 | 175179 | 299303 |  |
| South Central Pacific | Tuamotu | Tuam06 | 338346 | 296304 | 218218 | 189189 | 246246 | 352352 | | 319319 | 231251 | 147147 | 211227 | 345345 | 278278 | | 204206 | 167173 | 303303 |  |
| South Central Pacific | Tuamotu | Tuam07 | 330330 | 300308 | 218218 | 173183 | 246246 | 352354 | | 313327 | 265265 | 155169 | 191211 | 355355 | 276278 | | 206208 | 173175 | 303337 |  |
| South Central Pacific | Tuamotu | Tuam08 | 326326 | 296312 | 218218 | 187189 | 252252 | 352354 | | 313331 | 265265 | 169183 | 211223 | 345353 | 272278 | | 200200 | 171173 | 299303 |  |
| South Central Pacific | Tuamotu | Tuam09 | 342342 | 304308 | 218218 | 187191 | 252252 | 352352 | | 313329 | 207207 | 159177 | 191211 | 353359 | 278280 | | 206210 | 169179 | 293301 |  |
| South Central Pacific | Tuamotu | Tuam10 | 366366 | 304316 | 218218 | 173193 | 246246 | 352352 | | 331335 | 205233 | 171189 | 211227 | 349359 | 270278 | | 204206 | 175175 | 303303 |  |
| South Central Pacific | Tuamotu | Tuam11 | 302334 | 296308 | 216216 | 177183 | 249249 | 352352 | | 327327 | 243247 | 159175 | 191211 | 351353 | 270282 | | 200208 | 173179 | 301303 |  |
| South Central Pacific | Tuamotu | Tuam12 | 330338 | 308312 | 218218 | 183183 | 252261 | 352354 | | 313327 | 209209 | 147167 | 211211 | 345349 | 270278 | | 208212 | 173173 | 301301 |  |
| South Central Pacific | Tuamotu | Tuam13 | 330338 | 304308 | 218218 | 165193 | 246249 | 352354 | | 331331 | 209209 | 159181 | 219223 | 345359 | 270278 | | 206212 | 173175 | 299303 |  |
| South Central Pacific | Tuamotu | Tuam14 | 338338 | 304312 | 218218 | 189195 | 246255 | 352354 | | 313331 | 233259 | 147165 | 207207 | 345355 | 276280 | | 206206 | 169173 | 301303 |  |
| South Central Pacific | Tuamotu | Tuam15 | 330342 | 304308 | 212216 | 175189 | 264264 | 352352 | | 313313 | 209237 | 147147 | 211223 | 345353 | 280280 | | 206206 | 175175 | 303303 |  |
| South Central Pacific | Tuamotu | Tuam16 | 330350 | 308312 | 218218 | 189189 | 252252 | 352352 | | 317327 | 217269 | 151151 | 191227 | 345357 | 270278 | | 206206 | 169169 | 299301 |  |
| South Central Pacific | Tuamotu | Tuam17 | 334342 | 296300 | 218218 | 173183 | 255255 | 352352 | | 329335 | 207239 | 147189 | 211223 | 347361 | 270278 | | 200206 | 171179 | 293293 |  |
| South Central Pacific | Tuamotu | Tuam18 | 334346 | 300304 | 218218 | 173199 | 252270 | 352354 | | 311331 | 215231 | 159169 | 207227 | 351353 | 270270 | | 206212 | 173175 | 299303 |  |
| South Central Pacific | Tuamotu | Tuam19 | 338338 | 304308 | 218218 | 193193 | 249249 | 352352 | | 311319 | 261261 | 159169 | 211223 | 345359 | 266270 | | 212212 | 167173 | 291303 |  |
| South Central Pacific | Tuamotu | Tuam20 | 346346 | 304308 | 218218 | 187189 | 252258 | 352352 | | 311319 | 207265 | 177179 | 191223 | 357359 | 278280 | | 200204 | 169175 | 299303 |  |
| South Central Pacific | Tuamotu | Tuam21 | 322338 | 304308 | 216218 | 173183 | 246255 | 352352 | | 321327 | 249265 | 151169 | 211223 | 349359 | 278280 | | 200208 | 169173 | 293299 |  |
| South Central Pacific | Tuamotu | Tuam22 | 334338 | 304304 | 218218 | 175201 | 246252 | 352352 | | 311319 | 207225 | 147147 | 207223 | 345345 | 280280 | | 206210 | 173179 | 297299 |  |
| South Central Pacific | Tuamotu | Tuam23 | 330354 | 296312 | 216218 | 177191 | 264264 | 352354 | | 327333 | 231271 | 165177 | 211211 | 345345 | 270270 | | 200200 | 155177 | 301301 |  |
| South Central Pacific | Tuamotu | Tuam24 | 334338 | 304308 | 218218 | 193201 | 249249 | 352352 | | 331333 | 239241 | 169183 | 191207 | 345359 | 278278 | | 206212 | 171173 | 299303 |  |
| South Central Pacific | Tuamotu | Tuam25 | 342342 | 300304 | 218218 | 175193 | 249249 | 352354 | | 327327 | 207261 | 155171 | 191223 | 345351 | 274282 | | 200214 | 171175 | 301339 |  |
| South Central Pacific | Tuamotu | Tuam26 | 330354 | 296296 | 218220 | 173201 | 249249 | 352352 | | 313329 | 215233 | 155165 | 191207 | 345355 | 278278 | | 206212 | 169173 | 299303 |  |
| South Central Pacific | Tuamotu | Tuam27 | 322338 | 304308 | 216218 | 175187 | 249252 | 352352 | | 313319 | 207207 | 171183 | 207223 | 345353 | 272272 | | 200208 | 173175 | 299303 |  |
| South Central Pacific | Tuamotu | Tuam28 | 350350 | 308308 | 218218 | 165189 | 246255 | 352354 | | 319331 | 207213 | 161165 | 207211 | 357361 | 276276 | | 206212 | 173173 | 293303 |  |
| South Central Pacific | Tuamotu | Tuam29 | 354362 | 300300 | 218218 | 173195 | 252252 | 352352 | | 313327 | 239239 | 155181 | 191207 | 345359 | 270280 | | 200204 | 173173 | 299303 |  |
| South Central Pacific | Tuamotu | Tuam30 | 346350 | 308308 | 218218 | 183201 | 249252 | 352354 | | 319327 | 207233 | 175189 | 223223 | 355359 | 270270 | | 192200 | 169173 | 303303 |  |
| South Central Pacific | Tuamotu | Tuam31 | 354354 | 304308 | 216218 | 165189 | 252255 | 352352 | | 329331 | 265265 | 155161 | 207207 | 347361 | 270278 | | 206210 | 169175 | 301303 |  |
| South Central Pacific | Tuamotu | Tuam32 | 338338 | 308312 | 216218 | 173187 | 246246 | 352352 | | 321335 | 207239 | 155179 | 211211 | 353359 | 270270 | | 200206 | 169173 | 299299 |  |
| South Central Pacific | Tuamotu | Tuam33 | 342354 | 300304 | 218218 | 173173 | 249249 | 352352 | | 327335 | 217223 | 177177 | 211211 | 353359 | 272280 | | 206212 | 171171 | 301301 |  |
| South Central Pacific | Tuamotu | Tuam34 | 330354 | 296296 | 218220 | 173173 | 252252 | 352352 | | 313327 | 243247 | 155181 | 199199 | 353369 | 270280 | | 192210 | 173173 | 287287 |  |
| South Central Pacific | Tuamotu | Tuam35 | 350364 | 304308 | 218218 | 193193 | 252252 | 352352 | | 333337 | 239265 | 169177 | 207219 | 349359 | 272278 | | 204204 | 171173 | 293303 |  |
| South Central Pacific | Tuamotu | Tuam36 | 350364 | 304308 | 218218 | 165187 | 246255 | 352354 | | 313331 | 207233 | 163189 | 199211 | 345351 | 278280 | | 206206 | 173175 | 293303 |  |
| South Central Pacific | Tuamotu | Tuam37 | 362362 | 300304 | 218218 | 165173 | 246252 | 352354 | | 313313 | 213233 | 155163 | 207223 | 345347 | 278278 | | 208212 | 169175 | 279297 |  |
| South Central Pacific | Tuamotu | Faaite01 | 350354 | 296300 | 216218 | 173175 | 252252 | 352352 | | 327335 | 217221 | 177177 | 211211 | 353359 | 272280 | | 206212 | 171171 | 301301 |  |
| South Central Pacific | Tuamotu | Faaite02 | 338338 | 304308 | 216218 | 169183 | 252252 | 352352 | | 311327 | 243247 | 155181 | 199199 | 353369 | 270280 | | 192210 | 173173 | 287287 |  |
| South Central Pacific | Tuamotu | Faaite03 | 330346 | 300308 | 216218 | 191197 | 246252 | 352352 | | 333337 | 239265 | 169177 | 207219 | 349359 | 272278 | | 204204 | 171173 | 293303 |  |
| South Central Pacific | Tuamotu | Faaite04 | 330342 | 304304 | 216218 | 189189 | 252255 | 352354 | | 311331 | 207233 | 163187 | 199211 | 345351 | 278280 | | 206206 | 173175 | 293303 |  |
| South Central Pacific | Tuamotu | Faaite05 | 338358 | 304304 | 218218 | 183199 | 255261 | 352354 | | 311311 | 213233 | 155163 | 207223 | 345347 | 278278 | | 208212 | 169175 | 269297 |  |
| South Central Pacific | Tuamotu | Nengo01 | 346346 | 304308 | 218218 | 189193 | 255261 | 352352 | | 311317 | 207231 | 155159 | 191191 | 345353 | 270280 | | 200206 | 167173 | 303333 |  |
| South Central Pacific | Tuamotu | Nengo02 | 302330 | 296304 | 218220 | 165201 | 246255 | 352352 | | 317331 | 209209 | 159165 | 211211 | 345347 | 272278 | | 206210 | 173173 | 295301 |  |
| South Central Pacific | Tuamotu | Nengo03 | 330334 | 296304 | 218218 | 165171 | 252252 | 352354 | | 311327 | 247247 | 155185 | 191223 | 345353 | 278278 | | 204204 | 169173 | 269301 |  |
| South Central Pacific | Tuamotu | Nengo04 | 330354 | 304304 | 218218 | 193197 | 252252 | 352354 | | 317327 | 213213 | 181187 | 211223 | 353359 | 270272 | | 206210 | 167175 | 301301 |  |
| South Central Pacific | Tuamotu | Polynesie01 | 302338 | 304312 | 218218 | 183193 | 252252 | 352352 | | 327331 | 213225 | 151155 | 207211 | 347353 | 274278 | | 206212 | 173175 | 293303 |  |
| South Central Pacific | Tuamotu | Polynesie02 | 330338 | 300300 | 218218 | 171189 | 252255 | 352352 | | 327327 | 207231 | 147163 | 211223 | 353375 | 270278 | | 208210 | 173175 | 295303 |  |
| South Central Pacific | Tuamotu | Polynesie03 | 338338 | 308312 | 216218 | 183191 | 252252 | 352354 | | 311319 | 207237 | 155187 | 211223 | 349357 | 272276 | | 208210 | 173179 | 297299 |  |
| South Central Pacific | Tuamotu | Polynesie04 | 338338 | 308312 | 218218 | 183191 | 252252 | 352352 | | 311335 | 235239 | 147155 | 207211 | 353359 | 272276 | | 194206 | 167177 | 301347 |  |
| South Central Pacific | Tuamotu | Polynesie05 | 350354 | 296300 | 218218 | 183191 | 246255 | 352352 | | 311327 | 207239 | 147151 | 211211 | 345347 | 270278 | | 204212 | 171173 | 291303 |  |
| South Central Pacific | Tuamotu | Polynesie06 | 342346 | 296304 | 218218 | 183191 | 252255 | 352354 | | 323327 | 213217 | 151161 | 207223 | 345353 | 270278 | | 200208 | 169173 | 301301 |  |
| South Central Pacific | Tuamotu | Polynesie07 | 334338 | 300304 | 218218 | 181199 | 252255 | 352352 | | 311327 | 229229 | 147163 | 191211 | 359361 | 272280 | | 206208 | 173175 | 301303 |  |
| South Central Pacific | Tuamotu | Polynesie08 | 338338 | 308308 | 218218 | 171189 | 252261 | 352352 | | 319335 | 239265 | 147179 | 211211 | 347369 | 270278 | | 210212 | 169179 | 303303 |  |
| South Central Pacific | Tuamotu | Polynesie09 | 342354 | 304308 | 218218 | 171191 | 246246 | 352352 | | 311327 | 217239 | 147165 | 211223 | 347353 | 272276 | | 192212 | 173183 | 297301 |  |
| South Central Pacific | Tuamotu | Polynesie10 | 346358 | 300300 | 218218 | 171189 | 246252 | 352352 | | 319327 | 221247 | 147177 | 223223 | 345359 | 270278 | | 206212 | 175175 | 297301 |  |
| South Central Pacific | Tuamotu | Polynesie11 | 322326 | 304312 | 218218 | 173195 | 246252 | 352352 | | 311311 | 227229 | 155179 | 223223 | 345345 | 270278 | | 210210 | 169171 | 301303 |  |
| South Central Pacific | Tuamotu | Polynesie12 | 326338 | 300300 | 216218 | 171171 | 249252 | 352352 | | 327331 | 245265 | 147165 | 211223 | 349353 | 270280 | | 210212 | 169175 | 301301 |  |
| South Central Pacific | Tuamotu | Polynesie13 | 338346 | 304308 | 218218 | 171187 | 246249 | 352352 | | 311319 | 207229 | 147183 | 211211 | 353359 | 270278 | | 206206 | 167169 | 303303 |  |
| South Central Pacific | Tuamotu | Polynesie14 | 330330 | 304304 | 218218 | 173193 | 252255 | 352354 | | 327333 | 213235 | 171177 | 211223 | 353361 | 270270 | | 200206 | 171173 | 301303 |  |
| South Central Pacific | Tuamotu | Polynesie15 | 342346 | 304308 | 218218 | 171191 | 246252 | 352354 | | 331331 | 221265 | 151181 | 191219 | 349353 | 272278 | | 206212 | 169173 | 295301 |  |
| South Central Pacific | Tuamotu | Polynesie16 | 338342 | 296308 | 216218 | 183191 | 246249 | 352352 | | 311333 | 265265 | 151185 | 223223 | 345353 | 272278 | | 210212 | 167169 | 301303 |  |
| South Central Pacific | Tuamotu | Polynesie17 | 334354 | 300312 | 218218 | 189189 | 252255 | 352352 | | 311321 | 209209 | 159165 | 223223 | 349359 | 276278 | | 206206 | 167173 | 303303 |  |
| South Central Pacific | Tuamotu | Polynesie18 | 322342 | 304308 | 218218 | 173173 | 252255 | 352352 | | 333335 | 207213 | 155165 | 191227 | 345351 | 270278 | | 200212 | 173175 | 303303 |  |
| South Central Pacific | Tuamotu | Polynesie19 | 330338 | 308312 | 216218 | 171189 | 246252 | 352352 | | 311327 | 225247 | 147171 | 191207 | 355359 | 270278 | | 204204 | 169173 | 287301 |  |
| South Central Pacific | Tuamotu | Polynesie25 | 350350 | 304304 | 218218 | 183201 | 246255 | 352354 | | 327331 | 209265 | 155171 | 211211 | 353353 | 280280 | | 206212 | 173173 | 303303 |  |
| South Central Pacific | Tuamotu | Polynesie26 | 000000 | 300304 | 212218 | 169179 | 246258 | 352352 | | 311327 | 207227 | 159183 | 211223 | 345353 | 270278 | | 206216 | 171173 | 297303 |  |
| South Central Pacific | Tuamotu | Polynesie27 | 334338 | 300308 | 218218 | 165193 | 240252 | 352352 | | 311331 | 207233 | 173175 | 223223 | 000000 | 270278 | | 206206 | 167173 | 295303 |  |
| South Central Pacific | Tuamotu | Polynesie28 | 330350 | 296312 | 216218 | 183197 | 252261 | 352354 | | 327327 | 227233 | 147163 | 191207 | 345345 | 276280 | | 206210 | 167173 | 301303 |  |
| South Central Pacific | Tuamotu | Polynesie29 | 334342 | 296300 | 218218 | 169173 | 252270 | 352354 | | 327331 | 213237 | 159165 | 191223 | 353353 | 270270 | | 206214 | 169175 | 303303 |  |
| South Central Pacific | Tuamotu | Rangi01 | 350350 | 300312 | 218218 | 171187 | 252255 | 352352 | | 327331 | 223243 | 151155 | 211227 | 345355 | 278280 | | 206210 | 169175 | 301301 |  |
| South Central Pacific | Tuamotu | Raraka1 | 000000 | 304308 | 218218 | 165171 | 246252 | 354354 | | 327327 | 209241 | 157181 | 211223 | 345355 | 270280 | | 206206 | 173179 | 299303 |  |
| South Central Pacific | Tuamotu | Tapoto01 | 362370 | 304304 | 218218 | 195199 | 246252 | 352352 | | 327341 | 209265 | 167171 | 211223 | 353353 | 278280 | | 212212 | 171175 | 301303 |  |
| South Central Pacific | Tuamotu | Tapoto02 | 346350 | 304308 | 218218 | 193197 | 255270 | 352352 | | 319335 | 221235 | 155155 | 211223 | 353353 | 270278 | | 192206 | 169173 | 301303 |  |
| North Central Pacific | Tuvalu | Tuvalu01 | 362362 | 304308 | 216218 | 187193 | 246252 | 352354 | | 327327 | 217231 | 163167 | 207211 | 345353 | 278282 | | 204214 | 169173 | 297299 |  |
| North Central Pacific | Tuvalu | Tuvalu02 | 326350 | 296312 | 218218 | 165187 | 246252 | 352354 | | 331335 | 223223 | 151153 | 211211 | 345353 | 272276 | | 200208 | 167171 | 299301 |  |
| North Central Pacific | Tuvalu | Tuvalu03 | 342362 | 308308 | 218218 | 171187 | 252252 | 352354 | | 311327 | 221227 | 165173 | 211211 | 345359 | 270280 | | 202212 | 173175 | 269301 |  |
| North Central Pacific | Tuvalu | Tuvalu04 | 334362 | 304308 | 210218 | 175187 | 240252 | 352354 | | 311335 | 221245 | 157181 | 191227 | 353367 | 270278 | | 194208 | 169173 | 301303 |  |
